# Applied Proteomics in ‘One Health’

**DOI:** 10.3390/proteomes9030031

**Published:** 2021-06-30

**Authors:** Eleni I. Katsarou, Charalambos Billinis, Dimitrios Galamatis, George C. Fthenakis, George Th. Tsangaris, Angeliki I. Katsafadou

**Affiliations:** 1 Veterinary Faculty, University of Thessaly, 43100 Karditsa, Greece; elekatsarou@vet.uth.gr (E.I.K.); billinis@vet.uth.gr (C.B.); gcf@vet.uth.gr (G.C.F.); 2 Faculty of Public and One Health, University of Thessaly, 43100 Karditsa, Greece; 3 Hellenic Agricultural Organization DIMITRA (ELGO DIMITRA), 54248 Thessaloniki, Greece; galamatis@elog.gr; 4 Proteomics Research Unit, Biomedical Research Foundation of Academy of Athens, 11527 Athens, Greece; gthtsangaris@bioacademy.gr

**Keywords:** antibiotic resistance, biomarker, food safety, foodomics, One Health, pathogenesis, proteomics, veterinary, zoonotic infection

## Abstract

‘One Health’ summarises the idea that human health and animal health are interdependent and bound to the health of ecosystems. The purpose of proteomics methodologies and studies is to determine proteins present in samples of interest and to quantify changes in protein expression during pathological conditions. The objectives of this paper are to review the application of proteomics technologies within the One Health concept and to appraise their role in the elucidation of diseases and situations relevant to One Health. The paper develops in three sections. Proteomics Applications in Zoonotic Infections part discusses proteomics applications in zoonotic infections and explores the use of proteomics for studying pathogenetic pathways, transmission dynamics, diagnostic biomarkers and novel vaccines in prion, viral, bacterial, protozoan and metazoan zoonotic infections. Proteomics Applications in Antibiotic Resistance part discusses proteomics applications in mechanisms of resistance development and discovery of novel treatments for antibiotic resistance. Proteomics Applications in Food Safety part discusses the detection of allergens, exposure of adulteration, identification of pathogens and toxins, study of product traits and characterisation of proteins in food safety. Sensitive analysis of proteins, including low-abundant ones in complex biological samples, will be achieved in the future, thus enabling implementation of targeted proteomics in clinical settings, shedding light on biomarker research and promoting the One Health concept.

## 1. Introduction

**Figure 1:**
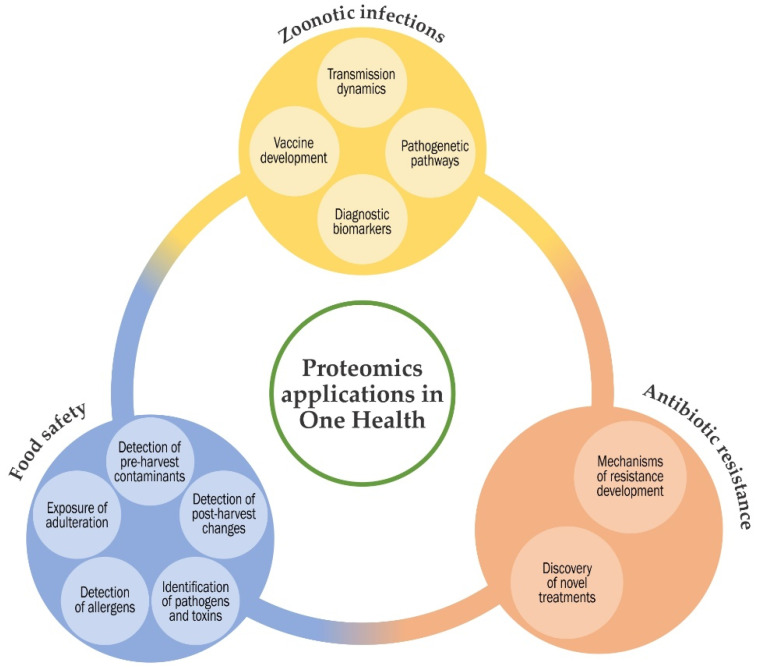
Summary diagram of the applications of proteomics methodologies in One Health.

Τhe concept of ‘One Health’ was developed at the start of the current century. One Health is the notion that the health of people, animals and ecosystems are strongly interconnected. The definition summarises the idea that human health and animal health are interdependent and bound to the health of the ecosystems in which they exist. The areas of work in which a One Health approach is particularly relevant, include the control of zoonoses, limiting bacterial resistance to antimicrobial agents and food safety [1] (Figure 1).

Controlling zoonotic pathogens at their animal source is the most effective way of protecting humans from the respective infections; consequently, strategies to control zoonotic pathogens must be developed to prevent animal and human diseases. Further, prevention of antibiotic resistance development at the animal level reduces the chances of dissemination of resistant strains to humans from animal sources. Indeed, the connection between animal health, food of animal origin and human health is more than evident for public opinion and consumers [2].

The proteome contains all proteins in a cell or tissue at any one time, thus taking into account all post-translational modifications. The proteome is dynamic and changes depending on various physiological and pathological conditions in the tissues of an organism. The purpose of proteomics methodologies and studies is to determine the proteins present in a sample of interest and to quantify changes in protein expression during various pathological conditions [3].

Proteomics includes a variety of technologies, which are divided in two major groups: the gel-dependent and the gel-independent methods [4,5,6,7]. Nevertheless, in both approaches, protein identification is performed with mass spectrometry (MS) [6]. In gel-dependent approaches, after isolation of the proteins by two-dimensional gel electrophoresis (2-DE), proteins are identified mainly by MALDI-TOF MS/MS (matrix-assisted laser desorption/ionisation-time of flight tandem mass spectrometry). In gel-independent approaches, the protein content of a sample is identified by MS [8]. Further, proteomics can be applied as bottom-up or top-down approaches [9]. In bottom-up proteomics, pure proteins or complex protein mixtures are subjected to proteolytic cleavage and the peptide products are analysed by MS. Ιn a top-down approach, intact protein ions or large protein fragments are subjected to gas phase fragmentation for MS analysis. Finally, data processing and evaluation are performed with bioinformatics approaches [10,11]. Detailed descriptions of proteomics techniques that can be used in such studies have been presented before [12,13] and are beyond the scope of this paper.

The objectives of this paper are to review the application of proteomics technologies within the One Health concept and to appraise the role of these methodologies in the elucidation of diseases and situations relevant to the One Health approach.

## 2. Proteomics Applications in Zoonotic Infections

Infections of animal origin that can be transmitted to humans are termed ‘zoonotic infections’ and pose worldwide risks to public health. Other infections that are mainly transmitted from person to person may also circulate in animals or have an animal reservoir, and may cause serious health emergencies (Table S1). These risks increase with globalisation, climate change and changes in human behaviour, giving pathogens numerous opportunities to colonise new territories and evolve into new forms [1].

### 2.1. Prion Zoonotic Diseases

Transmissible spongiform encephalopathies (TSEs) are fatal neurodegenerative disorders characterised by the formation of amyloid aggregates, vacuolation of brain tissue and accumulation in the Central Nervous System (CNS) of a pathological conformer (PrP^TSE^) of the host-encoded cellular prion protein (PrP^C^). There are various human or animal prion diseases; the human diseases include kuru, Creutzfeldt–Jakob disease and variant Creutzfeldt–Jakob disease, Gerstmann–Straussler–Scheinker syndrome and fatal familial insomnia; the animal diseases include bovine spongiform encephalopathy, scrapie, chronic wasting disease, transmissible mink encephalopathy, feline spongiform encephalopathy and ungulate spongiform encephalopathy [14]. Of the above, a One Health concern exists for bovine spongiform encephalopathy (for which confirmed cases in humans have been reported) and scrapie (for which so far there are only suspicions for transmission to humans) [15].

PrP^TSE^ has a central role in the pathogenesis of the disease, but other factors are likely also involved in the pathological process. The common pathogenetic event in TSEs is the conformational conversion of the host-encoded cellular prion protein (PrP^C^) into the abnormal form PrP^TSE^ [16]. PrP^C^ and PrP^TSE^ share the same primary structure and post-translational modifications [17], but differ in other technical characteristics, such as solubility—i.e., PrP^C^ is soluble but PrP^TSE^ has a strong tendency to form aggregates—and sensitivity to proteolytic treatment, i.e., PrP^C^ is promptly digested, whereas only a portion of PrP^TSE^ is degraded, yielding the protease-resistant core PrP27-30 [18]. Data implicate other non-PrP molecules as active participants in the misfolding process (‘interactome’), to catalyse and direct the conformational conversion of PrP^C^ or to provide a scaffold ensuring correct alignment of PrP^C^ and PrP^Sc^ during conversion. Such molecules may be specific to different scrapie strains to facilitate differential prion protein misfolding ATPase [19].

Proteomics has been used in order to elucidate the molecular pathogenesis of prion diseases of ruminants, with the aim to contribute to their early diagnosis by identifying relevant biomarkers [20,21] or to establish control strategies [22].

As protein–protein interactions are fundamental to all cellular processes, it should be possible to infer the function of PrP^C^ by identifying the proteins or ligands with which it interacts. Thus, characterisation of ‘prion interactome’ should also help to identify the possible contribution of other proteins in the conversion of PrP^C^ to PrP^Sc^ [23].

Brain tissue samples from sheep heterozygous to scrapie were evaluated using proteomics methodologies in order to identify polymorphisms of PrP that could be involved in the pathogenesis of transmissible encephalitis [24]. Molecules with high affinity for PrP^Sc^ from brain tissues of sheep were identified [25], whilst in an experimental scrapie model in hamsters, ferritin, calcium/calmodulin-dependent protein kinase a type II, apolipoprotein E and tubulin were identified as the major components associated with the protease-resistant core of PrP^TSE^ (PrP27-30), giving information on the cellular microenvironment of the pathological form of PrP [26]. Further, trace amounts of actin, cofilin, Hsp90a, the g subunit of the T-complex protein 1, glyceraldehyde 3-phosphate dehydrogenase, histones and keratins were also detected [26]. Some of these proteins (tubulin and ferritin) can bind to PrP [27,28], creating a disturbance of iron metabolism to cells due to the ability of iron to catalyse free radical formations that can destroy lipid membranes and other cellular constituents.

Alterations of iron metabolism seem to contribute to the development of neurodegeneration; some protective mechanisms against iron-induced oxidative damage may occur during the pathogenesis of TSEs. Iron is physiologically essential for growth and survival, playing important roles in many biological processes, such as electron and oxygen transport and DNA replication [29]. However, free iron can be toxic to cells due to the ability of iron to catalyse free radical formations that can destroy lipid membranes and other cellular constituents [30]. Kim et al. [27] showed a disturbance of iron metabolism in brains of scrapie-positive mice. Specifically, the expression levels of several proteins involved in iron metabolism, IRP1, IRP2, H- and H/L-ferritins, were significantly increased in the brain of scrapie-infected mice [27]. The increased iron content could result from the alteration of the blood–brain barrier caused by scrapie infection or from an increase in inflammatory cytokines, such as IL-*β* and TNF-*α* [31,32]. Those results together with previous findings suggest that disturbance of iron metabolism and related oxidative stress are closely associated with neurodegeneration in TSEs [27].

PrP^C^ is converted to the pathological isoform, PrP^Sc^. This has altered biochemical properties and forms amyloid aggregates that display infective characteristics. PrP^Sc^ is also the major component in biochemically enriched infectious samples [25]. The same authors also showed that amyloid aggregates contained components of the extracellular matrix (ECM) and proteins related to it, such as various types of collagen, proteoglycans (versican V3) and molecules related to them (BRAL1); additionally, components of the desmosomes (DSG1, JUP), ubiquitin, ferritin and CAMK2A were also identified [25]. These molecules correlated with prion infectivity and might participate in the pathogenesis of TSE.

Other proteins (calcium/calmodulin-dependent protein kinase alpha type II, Hsp90alpha) were associated with PrP^TSE^ fibrils in disease [26]. Apolipoprotein E co-localisation was found to occur in moderately mature lesions in prion diseases, where it contributes to the aggregation of PrP^sc^ after changes from cellular PrP isoform to PrP^sc^. ApoE is localised within PrP^TSE^ deposits [26], which supports the theory that it actively contributes to the pathogenesis of amyloid formation in prion diseases [33,34].

A biomarker for diagnosis of scrapie in sheep can be Na^+^/K^+^ ATPase [19]. This protein improved the effectiveness of disease-specific conversion of recombinant PrP, which suggests that it might act as a molecular cofactor. Finally, 9-aminoacridine compounds have been found to reduce the PrP^Sc^ burden [35]. Batxelli-Molina et al. [20] have suggested that possibly the use of a transthyretin monomer could help with diagnosis of the infection in early stages, when the causal agent disseminates from lymphoid organs into the brain.

### 2.2. Viral Zoonotic Infections

Proteomics studies of viral zoonotic organisms refer to investigations into rabies, coronavirus infections, West Nile virus disease and influenza virus infections.

#### 2.2.1. Rabies

Rabies is one of the longest-known infectious diseases in history. It is an acute, almost inevitably fatal, zoonotic neurological disease that can affect humans and other mammals. It is caused by rabies lyssavirus (family: Rhabdoviridae), which is transmitted from animals to humans primarily through bites [15]. Changes in the proteomes of the hippocampus, the brainstem and the spinal cord of infected dogs were evaluated by using 2-DE: 32, 49 and 67 proteins were differentially expressed between the three tissues. Then, quadrupole time of flight (QTOF MS) mass spectrometry and tandem mass spectrometry (QTOF MS and MS/MS) were used to identify these proteins. It was found that these included antioxidants (possibly protecting the CNS from oxidative stress), apoptosis-related proteins, cytoskeletal proteins (seen to downregulate, possibly as the result of CNS damage by rabies virus), heat shock proteins/chaperonins (possibly expressed as the result of a balance between tissue-damaging effects of the virus and the compensatory mechanisms of the host to cope with the infection), immune regulatory proteins, metabolic enzymes, neuron-specific proteins, transcription regulators, ubiquitination/proteasome-related proteins and vesicular transport proteins [36].

Among the above proteins, one (dihydropyrimidinase related protein-2) was found to be common in all three tissues and another fourtween were found to be common in two of the tissues—four common in hippocampus and brainstem (aconitase 2, annexin A6, guanine nucleotide-binding protein, mitochondrial inner membrane protein), four common in hippocampus and spinal cord (beta-globin, keratin 1, peroxiredoxin 2, pyruvate carboxylase) and six common in brainstem and spinal cord (alpha crystallin B chain, ATP synthase, creatine kinase B-type, heat shock protein 90kDa beta, N-ethylmaleimide sensitive fusion protein, silent information regulator 2). In the hippocampus, one protein network associated with gene expression, cellular development, growth and proliferation was observed, consistent with previous data of the alteration of the involvement of several genes in cell growth and proliferation [37,38]. In the brainstem, another protein network associated with drug and lipid metabolism was found, which included proteins related to the tissue damage caused by the infecting virus, as well as various novel proteins [38,39]. In the spinal cord, two protein networks were identified; one was associated with genetic disorder and small molecule biochemistry and the other related to neurological disease, energy production and nucleic acid metabolism [36].

In another study, proteome analysis of brain cerebral cortex from cats and dogs infected or not infected with the rabies virus were performed by means of 2-DE and two-dimensional fluorescence difference gel-electrophoresis (2-DIGE) methods. In total, 65 proteins were found with upregulation and 46 with downregulation. No differences were evident between dogs and cats and protein changes were similar in both species [40].

With regard to studies on identification of biomarkers, Thanomsridetchai et al. [36] proposed proteins that could predict the development of the clinical forms of rabies in affected dogs. These findings were employed by Mehta et al. [41], who worked in animal models and identified 10 proteins (proteoplipid protein 1, glutamate ammonia ligase, calcium calmodulin, dependent kinase 2 alpha, optic atrophy 1, hippocalcin-like protein 4, transgelin 3, adaptor-related protein complex 3, programmed cell death, interacting protein, limbic system associated membrane protein, karyopherin alpha 4). Thereafter, Mehta et al. [42] identified 143 proteins in the neuronal tissues of mice in response to infection and proposed that some molecules, e.g., KPNA4 [41], could be potential diagnostic markers for the disease. These were followed by work performed specifically in dogs, in which 36 proteins were found to be differentially regulated according to the form of the disease (paralytic or furious) [43]. In people, two proteins were validated as biomarkers (calcium calmodulin dependent kinase 2 alpha and glutamate ammonia ligase) for the diagnosis of paralytic rabies, in which both proteins were found to be upregulated [44].

#### 2.2.2. Coronavirus Infections

Coronaviruses are RNA viruses that can cause disease in mammals and birds. They belong to the order Nidovirales, suborder Cornidovirineae, family Coronaviridae and subfamily Orthocoronavirinae. Among domestic animals, coronaviruses can infect cattle (bovine coronavirus infection), pigs (porcine transmissible gastroenteritis [45], porcine haemagglutinating encephalomyelitis [46], porcine epidemic diarrhoea [47]), dogs (canine enteric coronavirus infection [48], canine respiratory coronavirus infection [49]) and cats (feline coronavirus infection, feline infectious peritonitis). The viruses can also cause disease in birds (e.g., infectious bronchitis [50]).

Seven species of coronavirus can cause infection in humans. Of these, four—specifically HCoV-E299 (*α*-CoV), HCoV-NL63 (*α*-CoV), HCoV-OC43 (*β*-CoV) and HCoV-HKU1 (*β*-CoV)—usually cause only a mild clinical disease. Three of these viruses are believed to be of animal origin: SARS-CoV, MERS-CoV and SARS-CoV-2, and can cause more severe diseases and risks to human health. With regard to SARS-CoV-2, it is considered that bats were the reservoir for this virus, although other research has indicated a potential role of Malaysian pangolins as hosts for the virus. Bats have been identified also as the reservoir for SARS-CoV [51] and diverse SARS-related coronaviruses [52,53]. It is noteworthy that since the beginning of the COVID-19 pandemic, transmission of the virus from humans to cats, dogs, tigers, lions and minks has been reported [1,54].

Proteomics applications were employed (Table S2) and have identified many post-translational modifications, which have led to many proteoforms and a broad heterogeneity of viral particles. Protein–protein interactions, protein abundances and post-translational modifications were studied and proteomics methodologies have contributed to the rapid detection of the SARS-CoV-2 virus by using MS proteotyping [55]. Comparative proteomics analysis was performed to compare the whole proteome sequences of SARS-CoV-2, bat CoV (SARS-like) and human SARS CoV by means of bioinformatics tools. Genetically, these three viruses are closely related, but nevertheless their protein sequence showed that the nucleocapsid (N) proteins were highly disordered, while other proteins (e.g., Nsp8, ORF6, ORF9b) were also disordered to a significant extent. Other proteins have shown lower disorder; almost all of these contained at least one intrinsically disordered protein region (IDPRs), thus shedding light onto the sequence and structural peculiarities and functions of the viral evolution [56]. In addition to these results, Xu et al. [57] found that ORF8 and ORF10 proteins in SARS-CoV-2 had no homologous proteins in SARS-CoV when comparing their proteomes. Finally, Chan et al. [58] noted that spike stalk S2 in SARS-CoV-2 was highly conserved and shared 99% identity with those of the two bat SARS-like CoVs (bat-SL-CoVZXC21 and bat-SL-CoVZC45) and human SARS-CoV.

Stukalov et al. [59] defined the interactomes of SARS-CoV-2 & SARS-CoV viruses and their potential effects on the influence on the transcriptome, proteome, ubiquitinome and phosphoproteome of a lung-derived human cell line. It was found that SARS-CoV-2 ORF8 was responsible for dysregulating the TGF-*β* pathway and the SARS-CoV-2 ORF 3 was responsible for dysregulating autophagy. The authors pointed out that there were various and many interactions of the phosphorylation and ubiquitination patterns on individual host proteins and detected 16,541 ubiquitination sites [59]; of these, 11,108 were differentially expressed in cases of SARS-CoV-2 or SARS-CoV infections. In contrast to infection by SARS-CoV, infection by SARS-CoV 2 led to phosphorylation of the antiviral kinase EIF2AK2 (PKR) at the critical regulatory residue S3328. This differential activation of EIF2AK2 could contribute to the difference in the growth kinetics between the two viruses. Most proteins of the virus were modified post-translationally. New post-translationally modified sites were found to be located at functional domains of viral proteins: ubiquitination was detected at SARS-CoV-2 N K338 and phosphorylation at SARS-CoV-2 and SARS-CoV N S310 and S311, respectively. A total of 27 proteins were detected in these viruses, of which 21 were ubiquitinated, with many ubiquitination sites being common to both viruses [59].

Messner et al. [60] developed a platform for ultra-high-throughput serum and plasma proteomics from people. Based on that, they identified 27 potential biomarkers for predicting the severity of COVID-19. Among these proteins, there were complement factors, the coagulation system, inflammation modulators and pro-inflammatory factors upstream and downstream of interleucin 6. The use of proteomics methodologies was a means to identify dysregulation in various coagulation factors in COVID-19 patients, accompanied by increased levels of anti-fibrinolytic components, among which various serine protease inhibitors (SERPINs) [61]. This contributed to the elucidation of the pathophysiology of the coagulopathic complications in patients with the disease.

In another study [62], the virus proteins were studied in detail; many (*n* = 332) different possible protein–protein interactions with host proteins were identified. Among the host proteins, 66 could be targeted by various drugs already licenced for use in humans [62]. In cases of infection, the host proteome was found to have undergone extensive modulation 24 h after infection and two protein clusters were identified. The first cluster included proteins downregulated during infection, mainly involved in cholesterol metabolism. The second cluster referred to the proteins that upregulated after infection, which included RNA-modifying proteins. This study showed also that splicing was an essential pathway for SARS-CoV-2 replication, as well as a potential therapeutic target in cases of disease [63]. The highlighting of cellular pathways can support the identification and characterisation of potential therapeutic interventions. In this case, the findings indicated that the spliceosome and the glycolysis inhibitors could be potential therapeutic agents for the treatment of COVID-19 [63].

#### 2.2.3. West Nile Fever

West Nile fever is caused by an RNA virus, in the genus *Flavivirus*. Most human infections remain asymptomatic, but West Nile fever (a mild flu-like fever) develops in approximately 20–30% of infected persons and West Nile neuroinvasive disease in <1%. The latter is characterised by encephalitis, meningitis, acute flaccid paralysis and even long-term neurological sequelae. Wild birds, horses and mosquitoes are involved in the transmission of the virus. *Culex* species, ornithophile mosquitoes attacking birds and also humans in periods of high humidity, has been identified as a principal vector of the virus and has an explosion-like replication. *Culex* species is the major vector of the virus in Europe [15,64,65].

Altamura et al. [66] have reported the inactivation of West Nile virus in spiked serum samples using SDS-PAGE (sodium dodecyl sulphate–polyacrylamide gel electrophoresis). These authors have shown its usefulness for identifying proteins differentially expressed in the serum of mice experimentally infected with the virus.

In a further study, LC (liquid chromatography) MS/MS was performed to investigate the phosphorylation events induced after infection with virus. Changes were found to 1657 phosphoproteins, of which, 12 h post-infection, 626 were upregulated and 227 were downregulated. These results were subjected to gene ontology analysis, which returned the inflammation-related spliceosome, ErbB, mitogen-activated protein kinase, nuclear factor kappa B and mechanistic target of rapamycin signalling pathways [67].

#### 2.2.4. Influenza Virus Infections

Influenza viruses are RNA viruses of the family Orthomyxoviridae that can cause a respiratory infection in mammals and birds. Based on group-specific antigens, influenza viruses are divided into A, B and C viruses. Only viruses in the A group are of zoonotic importance, as the only species in the genus *Alphainfluenzavirus*. Influenza A viruses are divided into subtypes based on two proteins on the surface of the virus: haemagglutinin (H) and neuraminidase (N); there are 18 different haemagglutinin subtypes and 11 different neuraminidase subtypes (H1 through H18 and N1 through N11, respectively). These viruses have been associated with many influenza outbreaks, the most recent of which occurred in 2009 and was a swine origin influenza A outbreak. Influenza viruses pathogenic for humans have determinants similar to those of strains of swine origin, which makes transmission possible between pigs and humans [68]. Avian influenza viruses may also be transmitted to mammals, especially pigs, which notably can be infected by both avian and human influenza viruses [69]. Dual infections can result in genetic recombinations of the viruses, which led to characterising pigs as ‘mixing vessels’ for influenza [15].

During proteomics evaluation of H1N1 influenza virus strains, differences have been detected in the interactions of the strains with animal hosts, particularly at macrophage level [70], findings that have provided information regarding pathogenesis of the disease. Using 2-DE and MALDI-TOF MS/MS, 13 proteins with upregulation and 21 with downregulation were detected, which were associated with molecular biosynthesis and heat shock proteins. Moreover, after inoculation of a human cell line, many molecular pathways were detected to have been affected; these included cell cycle regulation and lipid metabolism using quantitative proteomics [71]. The above findings have shed light on the virus biology and could contribute to establishing chemotherapeutic protocols for the infection. Moreover, protein changes in the virus can result in the development of mutations. The development of distinct sequences at the various nonamer positions can result in a large number of viral variants in the proteome, which would subsequently affect the dynamics of the population of the virus; for example, Abd Raman et al. [72] reported the mutational changes involved in the dynamics of H5N1 influenza virus.

There is also limited data after applying proteomics analysis for the avian species of the virus. Specifically, 38 proteins have been identified by using 2D-DIGE and MALDI-TOF MS/MS in the trachea of chickens challenged with strains IAV N9 [73]. Two annexin proteins (ANXA1, ANXA2) and a heat shock protein (HSPB1) were differentially expressed in infected chickens, which might contribute to the elucidation of pathogenetic mechanisms present during the infection [73]. In another study, Zou et al. [74] identified 18 proteins with upregulation and 13 with downregulation in brain tissue of chickens challenged with a pathogenic H5N1 strains, which has a confirmed neuropathogenetic activity. The differentially expressed proteins involved cytoskeleton proteins, proteins associated with the ubiquitin-proteasome pathway and neural signal transduction proteins; this way, it was possible to understand the interaction of the virus with the brain tissue of the affected birds and to reveal a possible mechanism for the neuropathogenesis of influenza [74].

Further, proteomics technologies were used in studies regarding interspecies reaction and how the virus adapts to humans from other species. When human cells were inoculated with the avian H7N9 nucleoprotein, proteomics analysis revealed that the spliceosome might be the most relevant pathway involved in the host response to the nucleoprotein expression [75]. In another study, H5N1, H9N2 and H1N1 strains from pigs were passaged in vitro to canine cells, revealing 12 proteins with upregulation and 49 with downregulation. These included cytoskeletal proteins, molecular biosynthesis proteins, ubiquitin-proteasome pathway proteins and heat shock proteins [76].

With regard to pathogenesis of the infection, Wu et al. [77] used proteomics technologies and reported that mast cells within infected hosts can support the replication of influenza A virus. In a different approach, Mitchell et al. [78] employed proteomics-generated data and presented two clusters of pathogenicity-related gene-expression. Further, in cases of co-infection with bacterial pathogens, Sender et al. [79] found that in the bronchoalveolar lavage of influenza virus-infected hosts, bacterial multiplication was more rapid due to the efflux of nutrients from capillary leakage into the alveolar space, as shown by quantitative proteomics methodologies (LC-MS/MS). A detailed review of the post-translational modifications identified in influenza by means of proteomics methodologies has been recently presented by Zhang et al. [80]. These have a significance in the pathogen-host interactions, as protein post-translational modifications can affect the virulence of the virus or the host-response; for example, protein phosphorylation events in lungs of infected mice can provide resources for the exploitation of the phosphorylation-mediated signalling network in the host cells [81]. Apart from the above studies, proteomics technologies were also used in studies of validation of vaccines, which are prepared from virus grown on embryonated chicken eggs, to be used for the prevention of influenza [82].

### 2.3. Bacterial Zoonotic Infections

Proteomics studies of bacterial zoonotic organisms refer to investigations into infections mainly with *Mycobacterium avium* subsp. *paratuberculosis*, *Mycobacterium bovis*, *Listeria monocyto* genes, *Bartonella henselae*, *Brucella* spp., *Burkholderia mallei*, *Campylobacter* spp., *Coxiella burnetii*, *Francicella tularensis*, *Salmonella* spp., *Borrelia* spp. and *Leptospira* spp.

#### 2.3.1. *Mycobacterium avium* subsp. *Paratuberculosis* Infection

*M. avium* subsp. *paratuberculosis* is the causative agent of paratuberculosis (Johne’s disease), a chronic intestinal inflammatory disease of ruminants. The infection has serious animal health implications and leads to significant economic losses in domesticated animals throughout the world [83]. The organism is suspected to be associated with Crohn’s disease of humans; the organism was recovered by cultural and molecular analysis in many, but not all, cases of the disease in people [15].

Hughes et al. [84], by using 2-D PAGE (polyacrylamide gel electrophoresis) and MALDI-TOF, identified 10 proteins that had upregulated expression in isolates of the organism recovered from the ileum of naturally-infected sheep. Some of these proteins such as ArgG and RocA may have a role in the adaptation of *M. avium* subsp. *paratuberculosis* to its niche and the utilisation of carbon sources therein. Then, Hughes et al. [85] presented the immunogenic effects of these proteins for potential inclusion in vaccines against the infection. Thereafter, Hughes et al. [86] showed proteomics differences between type I/III and type II strains of the organism (the two phenotypic classes of the bacterium). The use of proteins differentially present in the blood serum was suggested as a useful tool for the diagnosis of subclinical infection in small ruminants [87]. Zhong, Taylor et al. [88], using chromatographic techniques and MS/MS, also reported the same findings and have identified two biomarkers (transthyretin and *α*-haemoglobin) in the blood serum of sheep exposed to *M. paratuberculosis* subsp. *avium*.

Leroy et al. [89] have performed a large-scale post-genomic analysis of proteins of the organism, with a view to identify specific antigens-biomarkers that might possibly improve the diagnosis of the infection. These authors, by applying two complementary approaches, generated a final database [89] that represented the first established secretome of the organism and a useful source of potentially specific antigens. In total, 25 candidate diagnostic antigens were found. Of these, five proteins were tested in an ELISA (enzyme-linked immunosorbent assay) for their diagnostic potential on field serum samples; the combination of any three of these proteins provided a sensitivity of 94.7% and a specificity of 97.9% to the test, these being comparable to those of established, commercially available tests.

#### 2.3.2. Mycobacterium Bovis Infection

Tuberculosis is a chronic disease of humans and animals, caused by several pathogenic species of the genus *Mycobacterium*. Almost all of these can be transmitted between humans and animals, but nevertheless, only a small proportion of human infections (<5%) is of animal origin. The most important agent of zoonotic tuberculosis is *M. bovis*, the causal agent of tuberculosis in cattle. In the year 2008, the overall incidence of *M. bovis* tuberculosis in people in the European Union was 0.02 per 100,000 persons; in Germany, only 1.9% of all diagnosed cases of tuberculosis were attributed to *M. bovis* [15]. Nevertheless, other species, e.g., the European wild boars (*Sus scrofa*), can be infected with this organism and then transmit it to people [90].

With a view to establish accurate and financially viable tests for the infection in animals, Seth et al. [91] identified 32 host peptides that specifically increased in the blood of infected animals. A biologically significant protein, common to both tuberculosis and paratuberculosis in cattle was vitamin D-binding protein. The potential role of vitamin D in controlling the infection was elucidated by Liu et al. [92], who indicated that vitamin D expression via toll-like receptors 2/1 (TLR2/1) had led to increased intracellular of the organism through the induction of cathelicidin [92,93]. Moreover, Lamont et al. [94] detected 16 *M. bovis*-related peptides in the blood of infected cattle. Among these, vitamin D-binding protein showed the greatest sensitivity and specificity, whilst a *M. bovis* protein (polyketide synthetase 5) was also found to be useful for discriminating against infections by other mycobacteria.

Further, the pathogenetic mechanisms of *M. bovis* infection in animals and humans were studied by looking at proteomics changes in monocyte cell line (THP-1). In total, 2032 proteins were evaluated, among which 61 were found with differential regulation. These were involved in various pathways (e.g., the phagosome maturation pathway, the TNF signalling pathway). The findings provided an understanding of the pathogenesis of the infection, which could be further used in controlling animal-to-human transmission [95].

In extending the previous work, Lopez et al. [96] employed proteomics methodologies to analyse the leucocyte’ proteome in vaccinated and unvaccinated cattle. This study indicated for the first time the role of several defence pathways during the infection and the changes occurring in cattle vaccinated against tuberculosis. In total, 1222 proteins were seen differentially expressed, among them proteins related to kinase activity and receptor activity molecular functions, as well as extracellular, Golgi apparatus and endosome cell components (including complement factor C8 alpha and C8 beta, as well as toll-like receptors 4 (TLR4) and 9 (TLR9)). Specifically in vaccinated cattle, proteins of the Janus kinase (JAK)-signal transducer and activator of transcription and protein kinase C(PKC) signalling pathways were also identified, potentially involved in eliciting a response by vaccinated animals.

#### 2.3.3. *Listeria monocytogenes* Infections

Listeriosis is a microbial disease of animals and humans with a variety of clinical forms. Recent research has shown that the infections is primarily of foodborne origin, with animals themselves being occasional sources for the causal organism, *L. monocytogenes*. Outbreaks as well as most cases in individual people are caused after consumption of contaminated foods [97]. Such foods include raw milk and soft cheese, meat and meat products, raw smoked fish, mussels, vegetables, sprouts and salads. Pasteurisation of milk has been reported to contain the bacteria, in the cases that the initial load was very high, although it is also possible that post-pasteurisation contamination may also be important. Contamination with soil, dust or faeces is the result of insufficient hygiene during food processing. Milk may also be contaminated through *Listeria*-associated mastitis in animals, after haematogenous dissemination to the mammary gland or direct ascending infection [15].

Proteomics investigations have also revealed the thermal versatility of the causal organism and its adaptability to low temperatures. Protein synthesis and folding, nutrient uptake and oxidative stress pathways were the most important pathways involved in low temperature adaptation response. The relevant knowledge was important to evaluate the possibility of an intervention to counteract its growth at cold temperatures [98]. The adaptation processes affected biochemical pathways related to protein synthesis and folding, nutrient uptake and oxidative stress. Moreover, proteins implicated in energy-production metabolic pathways, e.g., glycolysis and Pta-AckA pathway, were present to a higher level in bacteria incubated at 4 °C, which indicates that cells show increased energy demand for growth in low temperatures. Generalising, this finding points out that proteomics methodologies may act as a significant means for elucidating mechanisms regarding the cold adaptation response [98]. Moreover, the differential expression of the proteins of three different strains of *L. monocytogenes* proteins during growth in the presence of high concentrations of bile salts indicated differences in the expression of cell-wall-associated proteins, DNA repair proteins, protein folding chaperones and oxidative stress-response proteins [99], indicating that the response to the various (micro)environmental conditions can vary among strains and serovars of the organism.

Proteomics techniques can contribute to the early detection of the organism. Conventional microbiological techniques need 4 to 5 days for the accurate detection of *L. monocytogenes*. With the use of MALDI-TOF MS identification, this time is substantially shortened and diagnosis can be achieved within 30 h after submission of samples [100].

#### 2.3.4. *Bartonella henselae* Infections

The genus *Bartonella* includes 20 subspecies, of which 10 are responsible for human infections. The most common syndromes caused by *Bartonella* organisms include cat scratch disease (*B. henselae)*, which is the most common *Bartonella* infection worldwide, Carrion’s disease (*B. bacilliformis*) and trench fever (*B. quintana*). As diagnostic techniques improve, the clinical spectrum resulting from *B. henselae* infection would widen further [101,102]. The reservoir of potentially infected animals includes primarily cats (*B. henselae*, *B. clarridgeiae*, *B. koehlerae*, *B. elizabethae*, *B. weissii*). *B. henselae* is transmitted among susceptible hosts by arthropod vectors [15,103].

The proteome of *B. henselae* was studied using 2-DE SDS-PAGE and MALDI-TOF-MS. Initially, a reference proteome map of the organism with 191 different proteins was produced. Heat shock proteins represent a major target of the human immune response in bacterial infections [104,105]. In this context, chaperonins were found to be reactive with serum from infected people in 2-DE immunoblots.

Then, 79 immunoreactive proteins were identified by using 2-DE SDS-PAGE and immunoblotting in serum samples of patients with *B. henselae* infections; of these, 11 proteins were considered to be useful for the serodiagnosis of the infection [106].

#### 2.3.5. *Brucella* spp. Infections

In small ruminants, *B. melitens* is is the cause of brucellosis, a significant abortifacient infection of these animals, and the main agent of human brucellosis. Contact with animals, occupational exposure and consumption of contaminated food (e.g., milk) are the main factors leading to infection of humans with the organism [107]. In early studies, the complete proteomic profile of *B. melitensis* was described [108]; this could be used as a reference to evaluate the virulence of strains of the organism. Later work involved the use of LC-MS (liquid chromatography-mass spectrometry) for the establishment of differences in the protein profile in the blood serum of sheep infected or not with *B. melitensis*; the aim was to distinguish naturally infected animals from vaccinated ones and thus to monitor the progress of vaccination campaigns and the national strategies to control the infection [109]. In this study, the numbers of peroxisome protein partners were found to be overexpressed only in the group of vaccinated animals. In previous theoretical studies, peroxisome proliferator-activated receptor *γ* Ligands had been found to enhance human B cell antibody production and differentiation [110], which may possibly be extended in sheep, thus indicating a facet of the protective effect of vaccination. Thereafter, Wareth et al. [111] studied the immunogenic proteins of *Brucella* in the blood of naturally infected animals and identified its immunodominant proteins, which included heat shock proteins, enzymes, binding proteins and hypothetical proteins; it was suggested that the bacteria expressed those proteins mainly for their survival against the host systems during infection [111].

*B. abortus* is the causal agent of brucellosis in cattle. Clinical symptoms of infected cows include abortion, reduced fertility and reduction in milk production. Transmission of the causal agent from cattle to humans can occur through direct contact with infected cows, their tissues (e.g., placenta) or dairy products contaminated with the agent. Recent studies have shown increased resistance of *B. abortus* to antimicrobial agents [112,113], leading to concerns regarding that resistance potentially resulting in treatment failure in infected people [114]. Proteomics analysis of *B. abortus* by 2-DE and peptide mass fingerprinting revealed that the differentially expressed proteins involved in membrane transport, particularly the high affinity amino acids binding proteins, and those involved in Sec-dependent secretion systems related to type IV and type V secretion systems, were differentially expressed; this difference was responsible for conferring specific host preference in the organism [115]. Wareth et al. [116] have reported that *B. abortus* field strains revealed 402 differentially expressed proteins, among which 63 were exclusively in the whole cell extracts of *B. abortus*. Comprehensive analysis revealed that 25 proteins of *B. abortus* were distinctly immunoreactive; dihydrodipicolinate synthase, glyceraldehyde-3-phosphate dehydrogenase, lactate/malate dehydrogenase and fumarylacetoacetate hydrolase proteins were reactive with the serum samples from all host species of the organism (i.e., cattle, sheep, goats, buffaloes) [116]. These proteins could be employed in serological assays of pan-*Brucella* antibodies. Moreover, immunoproteomics was applied to identify novel candidate proteins from *B. abortus* cell envelope (CE) for the development of a vaccine. In total, 163 proteins were identified (2-DE with MALDI-TOF MS and LC MS/MS), some of them related to outer-membrane protein (Omp) 25, Omp31, Omp2b porin, and 60 kDa chaperonin GroEL [117].

Pajuaba et al. [118] characterised a *B. abortus* S19 antigen preparation obtained by Triton X-114 (TX-114) extraction through immunoproteomics, with the aim to differentiate infected from vaccinated cattle. The proteomics characterisation revealed 56 protein spots, of which 27 were antigenic spots differentiating the seroreactivity profile between naturally infected and vaccinated animals. Moreover, MS/MS analysis identified five *B. abortus* S19 proteins (invasion protein B, Sod, Dps, Ndk and Bfr), which were related with antigenicity in naturally infected cattle. In a more recent study [119], non-homologous proteins to cattle and humans were selected for metabolic analysis. Only three membrane proteins (ABC transporter permease, acriflavine resistance protein B, penicillin-binding protein 2) were found to be potential candidates for inclusion in vaccines with cattle as the host, whereas one membrane protein (ABC transporter permease) was selected as novel drug target with humans as the host [119]. The results of this study could facilitate the discovery and release of new and effective drugs and would help designing and producing potent vaccines against the pathogen.

#### 2.3.6. *Burkholderia mallei* Infections

Glanders is a severe infection primarily of horses, donkeys and mules, caused by *B. mallei*. It is characterised by pustular skin lesions, multiple abscesses, necrotic processes in the respiratory tract, pneumonia and septicaemia. It is occasionally transmitted to humans, resulting usually in a fatal disease. Important reservoirs of the organism include horses, sheep, goats, dogs and large Felidae (e.g., lions, tigers). Appropriate health programs have eradicated glanders in many parts of the world; the last cases in Europe were recorded several years ago. Nevertheless, the disease is still present in many parts of Asia and Latin America [15].

A simple and rapid diagnostic tool was developed based by means of proteomics approach. Using immunoblotting with equine sera, 12 proteins were identified with diagnostic significance, some of which were immunoreactive proteins (e.g., GroEL, translation elongation factor Tu, elongation factor Ts, arginine deiminase, malate dehydrogenase, DNA directed RNA polymerase subunit alpha) [120]. GroEL was shown to be immunoreactive with antiserum produced from horses with glanders or mice challenged with *B. mallei* [121], findings that indicate the immunodominance of GroEL, as confirmed later by Dohre et al. [120]. Evaluation of recombinant GroEL protein was evaluated in an ELISA for diagnosis of the infection in horses revealed 96.0% sensitivity and 98.7% specificity [120].

Moreover, *B. mallei*’s whole-cell proteome was used to develop an immunoblotting assay for the serological diagnosis of the infection. Whole-cell proteome of the organism was prepared through sonication and the protein content was visualised by SDS-PAGE. A ladder pattern of the *B. mallei* immunoreactive antigens was clearly evident within the region of 20 to 90 kDa [122].

#### 2.3.7. *Campylobacter* spp. Infections

The many species of *Campylobacter* spp. can cause various acute to chronic infections in animals and humans. Direct or indirect transmission from vertebrate animals to humans has been documented for *C. jejuni*, *C. coli*, *C. lari*, *C. upsaliensis* and *C. hyointestinalis*. These species cause mild to severe gastrointestinal infections characterised by diarrhoea. The infections can disseminate to other systems of the hosts. Infections in humans occur primarily by ingestion of food contaminated with the bacteria, which includes raw or undercooked milk and poultry or pork meat [15].

Variations between two *C. jejuni* isolates, one strong and one poor coloniser, were evaluated using 2-DE and MALDI-TOF to identify differentially expressed proteins [Seal et al. 2007]. Three proteins (a branched outer membrane fibronectin (Fn) binding protein (CadF), putative serine protease (htrA) and a putative aminopeptidase (P)) were identified only in the strong coloniser, whilst a cysteine synthase and aconitate hydratase were detected only in the poor coloniser [123].

The immunogens of *C. jejuni* clone SA for sheep have been identified by studying infective strains of the organism, as well as blood samples from infected sheep. In total, 26 immunogenic proteins were detected, of which 8 were cytoplasmic proteins, 2 were cytoplasmic membrane proteins, 11 were periplasmic proteins, 3 were outer membrane proteins and 2 were extracellular proteins [124]. The major outer membrane protein was involved in solute transport across the bacterial cell wall and adhesion on the intestinal mucosa [125]. Recombinant MOMP provided >42% of the protective efficacy against intestinal colonisation in mice [126]. Outer membrane peptidePEB4 is an antigenic virulence factor implicated in host cell adhesion, invasion, and colonisation in *Campylobacter* [127]. VirB10, a structural protein in the outer membrane, of the type IV secretion system, was identified as immunogen in *C. jejuni* [124], but its suitability for possible use in vaccines was limited, given that only few strains of the organism recovered from cases of ovine abortion, had this particular protein [124], which may limit its potential. Asakura et al. [128] performed ex vivo proteomics analysis of *C. jejuni* in poultry, a main reservoir for human infections, after experimental challenge. By using, iTRAQ-coupled (isobaric tags for relative and absolute quantitation) 2-DE LC MS/MS analyses, 55 *C. jejuni* proteins were detected, among which 10 (FabG, HydB, CJJ81176_0876, MscS, CetB, FlhF, PurH, PgIJ, LpxC, Icd) showed upregulation within one week post-challenge, compatible with indicated fatty acid metabolism affecting bacterial adaptation to the chicken host.

O’Reilly et al. [129] reported that, after challenge with *C. jejuni*, an abundance of cytoskeletal proteins of the chicken small intestinal proteome, particularly actin and actin-associated proteins, increased over time. Villin-1, an actin-associated anti-apoptotic protein, was reduced, indicating that many of the changes in cytoskeletal protein abundance in the challenged birds were as a result of an increased rate of apoptosis. Proteins associated with metabolism, energy and TCA cycles and glycolysis and membrane transport (HSP70, HSP710 and HSP108) were found to be reduced over time in the intestine of birds challenged with the organism [130,131,132,133].

Ayllón et al. [134] have used a comparative proteomics approach to identify the mechanisms involved after *C. jejuni* infection of its hosts. Human and porcine intestinal cell lines were infected with *C. jejuni* for up to 24 h. Proteomics analysis indicated significantly regulated biofunctions in human cells, related with engulfment and endocytosis, and supported by pathways related to infection, for example caveolar- or clathrin-mediated endocytosis signalling. In porcine cells, the same techniques were applied and inflammatory response and signalling pathways controlling cellular functions, such as cell migration, endocytosis and cell cycle progression, were found to be downregulated. These differences in the response to infection were supported by the different pattern of adhesion and invasion proteins expressed by *C. jejuni* in human and porcine cells. No marked differences in expression of virulence factors involved in adaptive response and iron acquisition functions were observed.

Binding-related proteins were detected on the skin of chickens by two-dimensional overlay assay and liquid chromatography mass spectrometry (LC-MS); chicken serum albumin (CSA) was identified as the most significant among these. Moreover, using the same approach, flagellar hook protein E (F1gE) and major outer membrane protein detected in *C. jejuni* were identified as bacterial adhesins binding the CSA [135]. The ability to bind CSA was also confirmed using recombinant F1gE and MOMP of *C. jejuni* [Taniguchi et al. 2021]. These findings suggest that adhesins expressed on *C. jejuni* bacteria may bind specifically to the hosts through proteins present on their skin.

#### 2.3.8. *Coxiella burnetii* Infections

The intracellular organism *C. burnetii* is the cause of Q fever. In animals, the infection can cause abortion, although it is usually subclinical. *C. burnetii* is excreted in vaginal secretions, aborted material, milk, faeces and urine [136,137,138]. Human infections have been recorded mainly after consumptions of milk of affected animals, as was the case in an outbreak of the infection in the Netherlands, which occurred subsequently to an outbreak in goat herds [139,140].

With the application of proteomics technologies, bacterial protein fractions enriched for outer membrane proteins have been detected. These were considered to be important for the development of new vaccines, due to their exposure to host immune cells [141]. Hence, use of these vaccines can limit the bacterial loads in animals and consequently, the risk of human infection will be decreased.

#### 2.3.9. *Francicella tularensis* Infections

Tularaemia is a contagious disease affecting the lymph nodes in various animal species. In some populations of wild-living rodents and lagomorphs, it may also cause septicaemia and epidemic disease. The infection is transmissible from animals to humans. In humans, the infection may remain asymptomatic or lead to clinical signs, from skin ulcers with regional lymphadenopathy to severe pleuropulmonary and typhoid-like generalised illness [15].

Mucha et al. [142] identified candidate proteins for a vaccine against the infection, based on the identification of proteins necessary for adhesion of the organism onto endothelial cells. Thereafter, an immunoproteomics approach was employed, based on the techniques of 2-DE and immunoblotting combined with MS, for the elucidation of immunogenic components and potential recognition of vaccine candidates. Whole-cell soluble protein extract of *F. tularensis* was separated by 2-DE and immunoblots were developed with sera raised in rabbits after their immunisation with the organism. That led to recognising 28 immunoreactive proteins after performing MS/MS. Then, the rabbit immunoproteome of *F. tularensis* was compared with those previously reported and, of the above proteins, 12 were recognised by human serum [143]. Nine proteins were found to be immunogenic in rabbits, mice and humans; of these, eight were new ones. The immunoreactive proteins identified in the study may be used in the design and development of protein subunit vaccines against the disease [143].

#### 2.3.10. *Salmonella* spp. Infections

*S. enterica* serovar Typhimurium (*S. typhimurium*) is one of the most frequent *Salmonella* serotypes recovered from samples from pigs, the species in which it is typically carried. It is a significant cause of an acute food-borne infection of humans, human enterocolitis [144]. *S. enterica* serovar Enteritidis is the predominant agent causing salmonellosis in poultry [145]. The organism is a food-borne pathogen for humans, mainly through contamination of eggs and egg products.

Arce et al. [146] have used proteomics technologies (2-DIGE) in samples of intestinal mucosa of pigs infected with the organism, in order to better understand the pathogenesis of infection and the pathophysiological pathways involved after infection. In total, 44 different proteins were recorded with significant activity. The analysis indicated that keratins and the intermediate filaments could play an important role in the damage of the intestinal mucosa and in the establishment and promotion of the infection. Samples of mesenteric lymph nodes were also assessed and the proteome response indicated an association with the induction of processes, such as phagocyte infiltration, cytoskeleton remodelling and pyroptosis [147]. In another study, proteome evaluation in pigs infected with *S. enterica* serovar Typhimurium revealed 51 proteins that were involved in the immune response of the host, the apoptosis and pathogen-mediated cell invasion, thus indicating the modulation of host responses in vivo [148]. In pigs, during infections by *Salmonella enterica* serovar Thyphimurium, there is an increase in the abundance of cytoskeletal proteins in the intestinal proteome [148]. In a later study, the same authors reported that a higher number of changes in protein expression was quantified in the ileum, with protein changes referring to proteins involved in inflammatory tory response or connective tissue disorders. In the colon, protein changes referred to those involved in cell death and survival, tissue morphology or molecular transport at the early stages and tissue regeneration [149].

Similar results have been found after experimental infection of poultry with *S. enterica* serovar Enteritidis. In that case, specific upregulation of mucin was particularly evident [150]. This increased mucogenesis has been considered to be a T cell-dependent inflammatory process [151], with severe infiltration of T cell subpopulations into the intestinal mucosa. Moreover, increased density of mucin layer in the intestine, especially at the caecum (the main site of *Salmonella* colonisation), might be a result of multiplication of the bacteria in that region and the severe inflammatory response taking place [152]. Polansky et al. [153] reported the changes in protein abundance in the liver and blood serum in response to *S. enteritidis* infection, using shotgun proteomics. Complement and coagulation cascades, TNF (tumour necrosis factor) signalling, antigen processing and presentation were activated in the liver following infection with *Salmonella* Enteritidis. Chicken proteins that decreased in the liver were involved in glycolysis, the citrate cycle, oxidative phosphorylation and fatty acid metabolism. No functional category was significantly activated or suppressed in the serum, indicating a local reaction in the intestine only. Differently abundant proteins characterise the bird’s response to infection and could be also used as markers of their health status. In order to identify antigenic serovar Enteritidis outer membrane proteins (OMPs) that could be employed for subunit vaccine development, a proteomic map of the antigenic OMPs of serovar Enteritidis was presented by using 2-DE methodology [145].

The proteins expressed by chicken CD4^+^, CD8^+^ and *γδ* T-lymphocytes from the spleen after infection with serovar Enteritidis were described by Sekelova et al. [154]. Inducible proteins in CD4^+^ lymphocytes included ribosomal proteins and downregulated proteins localised to the lysosome. CD8^+^ T-lymphocytes induced MCM (minichromosome maintenance) complex proteins, proteins required for DNA replication and machinery for protein processing in the endoplasmic reticulum. Proteins inducible in *γδ* T-lymphocytes referred to immunological response and oxidative phosphorylation [154] and can be used as markers specific for each lymphocyte subpopulation.

#### 2.3.11. *Borrelia* spp. Infections

Borreliosis (Lyme disease), caused by *B. burgdorferi sensu lato*, develops in animals and humans with a variety of clinical signs. The most important reservoirs of the organism are wild rodents, particularly wood and yellow necked mice, bank voles and hedgehogs, whose complement does not affect the bacteria, in contrast to the domestic animals, the complement of which lyses borreliae. The primary vectors of *B. burgdorferi s.l.* are various hard ticks that feed on the hosts for several days [15].

With regard to study of the organisms, MS was performed in bacterial isolates and in total, 30 bacterial proteins were identified [155], helping to develop subunit vaccines containing various antigens associated with effective adjuvant and delivery systems of antigens.

*Borrelia* was found to bind to a host’s complement regulatory factor H (fH) to evade complement attack. However, binding affinities between fH-binding-proteins (FHBPs) of Borrelia and fH from various hosts are disparate. Experiments performed to unfold the underlying molecular basis of this disparity revealed that recombinant BbCRASP-1 (major FHBP of *B. burgdorferi*) neither interacted with sushi 6–7 nor with sushi 19–20 domains of fH in cattle and pig, but showed binding affinity to both sushi domains of human fH, sushi 6–7 of mouse and sushi 19–20 of sheep. Further, peptide-spot assay revealed three major binding sites (sushi 6:(335–346), sushi 7:(399–410) and sushi 20:(1205–1227)) in human fH that can form BbCRASP-1:fH interface, while (HENMR341)-H-337 residues in sushi 6 are crucial for rigid BbCRASP-1:fH complex formation. Amino acid stretches DTIEFTCRYGYRPRTALHTFRTT in ovine sushi 19–20 and SAYWEKVYVQGQ in mouse sushi 7 were important sites for fH:BbCRASP-1 interaction. Comparative analysis of the amino acid sequences of sushi 6 of cattle, pig and human revealed that bovine and porcine fH lack methionine and arginine in HENMR pocket, which may impede the formation of fH:BbCRASP-1 interface [156].

After proteomics network analysis, 15 proteins were selected, which were then subjected to bioinformatics analysis to predict their antigenic properties. Based on the strategy applied in this study, the proteins encoded by erpX (ErpX proteins, UniProt ID: H7C7L6), erpL (ErpL protein, UniProt ID: H7C7M3) and erpY (ErpY protein, UniProt ID: Q9S0D9) were suggested as a novel set of vaccine targets to control Lyme disease [157].

#### 2.3.12. *Leptospira* spp. Infections

Leptospirosis is considered the most widespread zoonotic disease in the world. Its aetiological agents are bacteria of the genus *Leptospira*. Humans may be exposed to *Leptospira* spp. as a result of direct or indirect contact with infected animals or through contaminated environment (e.g., water). Various vertebrate animals, e.g., rodents and cattle, act as carrier or reservoir hosts of the organism. People working with animals (e.g., farmers, meat-workers) are at higher risk of infection. Clinical signs of the infection vary and include jaundice, reproductive disorders, agalactia, neonatal problems and uveitis [15]. The existence of over 250 serovars of the organism and the limited knowledge regarding pathogenesis of the infection have hampered the development of diagnostic tests using biomarkers [158].

In various proteomics studies, the virulence factors of *Leptospira* were targeted, with the aim to develop biomarkers for diagnosis of the infection and to promote the manufacturing of relevant vaccines. In particular, comparisons were made between *L. interrogans* and *L. canicola* [159], *L. copenhageni* [160,161], *L. pomona* [162], *L. lai* [163,164,165], *L. australis*, *L. bratislava* and *L. autumnalis*, as well as between *L. icterohaemorrhagiae* and *L. biflexa* [166]. Identification of the proteins and the differences in protein profiles between the species of the organism reflect their antigenicity, which can be useful in the development of vaccines. The outer membrane protein OmpL1, lipoproteins LipL32, LipL36, LipL41 and LipL48, leptospiral OmpA-like protein Loa22 and leptospiral immunoglobulin-like protein LigA and LigB in many pathogenic *Leptospira* spp. isolates [160,167,168,169,170,171,172,173] have a greater composition and structural complexity than those in isolates with intermediate pathogenicity or in non-pathogenic isolates [174]. A number of these proteins bind to extracellular matrix components, such as collagen, fibronectin, laminin or plasminogen, for adhesion, penetration or colonisation of the host tissues and development of pathogenetic action [175]. Moreover, bacterial proteins of the bacterial surface (‘surfaceome’) [176] were identified by means of a proteomics approach in an effort to understand mechanisms of host-adaptation, pathogenicity and development of relevant vaccines.

#### 2.3.13. Other Infections

Extensive proteomics studies have been performed in bacteria, causal agents of mastitis, which had been recovered from cases of the infection in cattle, sheep or goats. These have included *Staphylococcus aureus* [177,178,179], *Staphylococcus epidermidis* [180], *Streptococcus uberis* [181,182,183], *Escherichia coli* [184,185], *Mannheimia haemolytica* [186,187] and *Mycoplasma agalactiae* [188]. These studies have been extensively reviewed in other recent relevant publications [13,189].

### 2.4. Protozoan Zoonotic Infections

Proteomics studies of protozoan zoonotic organisms refer to investigations into *Cryptosporidium parvum*, *Toxoplasma gondii*, *Giardia* spp. and *Leishmania* spp. infections.

#### 2.4.1. *Cryptosporidium parvum* Infections

Members of the genus *Cryptosporidium* are the causal agents of a significant and financially important diarrhoeic disease of young ruminants, and they may also cause disease in people, which can be very severe in immunocompromised people [15]. *Cryptosporidium* may be found in soil, food, water or surfaces that have been contaminated with faeces from infected animals. Humans are infected by the oral route, either after touching contaminated surfaces or by ingesting contaminated food. Cryptosporidium is the leading cause of waterborne human infections [15].

Snelling et al. [190] have performed LC-MS/MS coupling with a stable isotope N-terminal labelling strategy using iTRAQ reagents on soluble fractions of sporozoites (the infective stage of the protozoon) of both non-excysted (transmissive) and excysted (infective) sporozoites (i.e., sporozoites inside and outside oocysts). Shotgun proteomics was also performed on insoluble fractions from non-excysted and excysted sporozoites. In total, 303 proteins of the organism were identified, 56 of which were detected for the first time, although they had been previously described on theoretical basis. The expression of 26 proteins increased significantly during excystation. These included ribosomal proteins, metabolic enzymes and heat shock proteins. These proteins would be potential targets for effective anti-protozoan drugs and also possibly vaccine targets, as they would block parasite entry into host cells.

Further, a comprehensive analysis of the proteins of an oocyst/sporozoite preparation of *C. parvum* was presented [191]. Three proteome platforms were employed and in consequence 4800 individual proteins were identified, corresponding to 1237 non-redundant proteins, amounting to about 30% of the predicted proteome of the parasite. Peptide data were also mapped and a database for the proteome data was developed (http://cryptodb.org) to support further studies related to the organism. It was hypothesised that the expression of proteins was likely associated with the invasion and intracellular establishment of the parasite [191]. Comparison of the expressed proteome with existing transcriptional data revealed only a weak correlation, indicating limitations on the current knowledge about the biology of this protozoan parasite.

#### 2.4.2. *Toxoplasma gondii* Infections

Toxoplasmosis is a systemic disease occurring worldwide in humans and animals after infection with *T. gondii*. Cats and other Felidae are the final hosts of the parasite, and, therefore, play a central role in its transmission. Sheep and goats are the animals mostly affected by the organism, species in which the parasite can cause abortion and foetal or neonatal death [192]. Most horizontal transmissions to humans are caused by ingestion of one of the two persistent stages of *T. gondii*, i.e., tissue cysts in infected meat or meat products or oocysts in food or water contaminated with feline faeces [15,193]. *T. gondii* is an intracellular protozoan parasite, tissue cyst-forming coccidian that secretes various proteins, including kinases, to manipulate host cell responses.

The proteome of the wall and sporocyst/sporozoite fractions of mature, sporulated oocysts were characterised using one-dimensional gel electrophoresis followed by LC-MS/MS on trypsin-digested peptides, elucidating the molecular pathogenetic pathways of the infection. In total, 1021 proteins were identified in the sporocyst/sporozoite fraction and 226 were identified in the oocyst wall fraction [194]. Among these, 172 proteins were identified for the first time; some of them were involved in conferring environmental resistance, among which there was a family of small, tyrosine-rich proteins present in the oocyst wall fractions and late embryogenesis abundant domain-containing (LEA) proteins in the cytosolic fractions [194].

Host–parasite interactions due to phosphorylation of host cell proteins kinases enhance virulence and maintenance of infection. Al-Bajalan et al. [195] performed a study in which proteome-wide phosphorylation events of host cell proteins were investigated after *T. gondii* infection, followed by pathway analysis on host signalling pathways. They found that about one-third of the phosphoproteomes, approximately 21% of the phospho-motifs and several pathways, e.g., glycolysis/gluconeogenesis and mTOR pathways of the host cell, were differentially enriched between infection with *T. gondii* and a closely related intracellular protozoan parasite and tissue cyst-forming *Neospora caninum* [195].

#### 2.4.3. *Giardia duodenalis* Infections

Giardiosis is caused by the ubiquitous flagellated protozoon *G. duodenalis* (*G. lamblia* or *G. intestinalis*). *Giardia* infections are common worldwide in lambs, calves (a species in which incidence rate up to 100% can be observed) and piglets. In dogs and cats, infection rates reach 2–15% and can be higher in young animals. Clinical disease is rare. The greatest risk of zoonotic transmission appears to be from companion animals, e.g., dogs and cats, although further studies are required in different endemic foci in order to determine the frequency of such transmission. Zoonotic sub-assemblages of the parasite have been frequently found [196,197].

A combination of proteome and genome data has been used to identify unique basal body proteins of the protozoan parasite [198], identifying 75 homologues of conserved basal body proteins in the genome. Most of these proteins are localised to additional cytoskeletal structures in interphase trophozoites, which might possibly explain the roles of the flagellae and specific organelles in the motility of the parasite [198]. Proteomics analysis has also been used to study metabolism in mitosomes, to increase understanding of the function and evolutionary origin of these organelles. It was shown that the small proteome of the mitosome reflected the reduction in mitochondrial metabolism, limited to the FeS cluster assembly pathway [199]. Furthermore, proteomics analysis of ventral disc extracts and comparison with the genome database of the organism has been used to identify novel proteins associated with the ventral disc and lateral crest. These structures are considered to be critical for attachment of the parasite onto the host, as the trophozoites attached onto the intestinal epithelium by means of the microtubule structure, termed ‘ventral disc’, and its surrounding structure forming a continuous perimetric seal with the substrate, termed ‘lateral crest’. All these have contributed to understanding the pathogenetic action of the parasite [200].

The proteomic profile of soluble and insoluble protein fractions of *Giardia* trophozoites was analysed by 2-DE. The proteomic map of soluble and insoluble protein fractions led to the identification of 187 proteins. Most of these proteins (82) were classified as metabolic proteins, mainly associated with carbon and lipid metabolism, including 53 proteins with catalytic activity. Some further proteins corresponded to antigens, while others were related to virulence [201].

#### 2.4.4. *Leishmania* Infections

Leishmaniosis is caused by protozoa of the genus *Leishmania*, which are transmitted by sandflies (*Lutzomyia* and *Phlebotomus*). The disease is presented with a wide clinical spectrum, depending on the protozoan species involved and the genetic background and immunological status of the host. There are three main clinical forms of the infection: visceral (the most grave form), cutaneous (the most common form) and mucocutaneous [15,202].

Most proteomics studies were aiming to identify targets for vaccine development or to investigate drug-resistance mechanisms. Moreover, virulence studies have also been performed, which could contribute to identification of potential drug targets and immunotherapy [202]. Quantitative proteomics methodologies have been used to determine protein expression levels between varying life stages of *Leishmania* spp.; specific strains resistant and sensitive to various anti-leishmaniasis drugs, as well as the interactions between the protozoa and their hosts have been evaluated. Specifically, an isotope-labelled (isotope-coded affinity tag, ICAT) quantitative proteomics approach was used to quantify differentially expressed proteins in differing life stages of *L. infantum*, thus, identifying 62 differentially expressed proteins [203]. Another quantitative analysis of proteins by isobaric tags for relative and absolute quantitation (iTRAQ) was applied to study the phosphoproteome modulation in *Leishmania* [204]. Further, large-scale proteomics on cytoplasm and membrane enriched proteomes from *L. infantum* amphotericin-resistant or -sensitive strains were employed to detect 97 differentially expressed proteins [205], which could further explore the mechanisms of *L. infantum* drug resistance.

*Leishmania* spp. proteoforms have been also studied. These may be the results of differences in mRNA splicing, polymorphisms of single amino acid, shifts of reading frame or post-translational modifications; thus, *Leishmania* spp. can show further biological functions [202]. Proteoform mapping was based mainly on the differential mobility on 2-DE. The proteome of a strain overexpressing trypanothione reductase, coding for an enzyme involved in the metabolism of trypanothione (a trypanosomatid exclusive reduced thiol involved in redox control) was analysed by 2-DE followed by MALDI-TOF MS [206]. Four differentially regulated spots were associated with different proteoforms of trypanothione reductase based on similar molecular weight and change in pI (isoelectric point) [206]. Another study reported the modulation of HSP90 proteoforms using 2D-DIGE (two-dimensional difference gel electrophoresis) coupled with MALDI-TOF MS of TiO_2_-enriched phosphoproteome of *L. donovani* stages [207]. Other studies involving 2-DE separation and MALDI-TOF and MS/MS were employed for the identification of phosphoproteins from *L. donovani* [208]. Some proteoforms were stage-specific and a great majority showed modifications other than phosphorylation. The detection of specific leishmanial proteoforms applied in different pathophysiological states can offer specific targets for chemo and immunotherapy.

Proteomics have been also used to study the post-translational modifications (PTMs) of the protozoon [207]. PTMs are involved, among others, in several cellular processes in trypanosomatids, including adherence, invasion and evasion of host cells, regulation of immune response, survival inside the vector and transition between life stages. The quantitative mapping of the PTMs of *Leishmania* spp. has significant potential for the discovery of putative drug targets and/or vaccine candidates [202]. For example, proteomics was used in the investigation of differentially expressed secreted proteins of amphotericin B-sensitive or -resistant isolates of *L. donovani* by applying label free quantitative LC-MS/MS approach, with totally 406 differentially expressed proteins finally identified [209]. Further, after protein classification according to biological process, the identified upregulated proteins in resistant parasites were involved in molecular pathways, e.g., carbohydrate metabolism, stress response, transporters and proteolysis. These provided the metabolic pathways of resistant parasites, which further unravelled an adaptive mechanism for *L. donovani* [209].

Proteomics were also used for the detection of biomarkers to improve diagnosis of the disease. Ejazi et al. [210] have screened urine-reactive leishmanial membrane proteins as potential biomarkers, specifically elongation factor lei (EF1-alpha), alpha-tubulin and glycoprotein 63. Franco-Martinez et al. [211] identified a total of 169 proteins in the blood proteome of dogs with leishmanial infection; C8 alpha chain, adiponectin, transferrin, sphingomyelin phosphodiesterase acid-like 3A and immunoglobulins showed different modulation between the stages of the infection and could be considered and further validated for the early diagnosis of the infection.

Agallou et al. [212] conducted a comparative immunoproteomics analysis of *L. infantum*, aiming to identifying molecules of protein extracts from late-log phase L. infantum promastigotes recognised by antibodies of sera from asymptomatic and symptomatic dogs. In total, 42 protein spots were found to differentially react with IgG from asymptomatic hosts and could be used as candidate antigens for vaccine development. Subsequently, a chimeric multi-epitope protein composed of multiple CD8^+^ and CD4^+^ T cell epitopes was designed in order to develop a novel vaccine against the protozoon [213].

### 2.5. Metazoan Zoonotic Infections

Proteomics studies of metazoan zoonotic organisms refer to investigations into *Ancylostoma caninum*, *Angiostrongylus cantonensis*, *Trichinalla spiralis* and *Echinococcus* spp. infections.

#### 2.5.1. *Ancylostoma caninum* Infections

*Ancylostoma caninum* is a nematode, which principally infects dogs, localising in the small intestine. Humans often become infected with direct transmission, with larvae having accessed through the skin, for example, if people walk barefoot on soil contaminated with larvae of the parasite. Eosinophilic enteritis is the consequence of infection and in humans it is also associated with atopic disturbances and food allergies.

Using a combination of techniques, SDS-PAGE and OFFGEL electrophoresis, in combination with mass spectrometry, Morante et al. [214] analysed the parasite’s excretory-secretory products (ESP). In total, 315 proteins were detected in the ESP, of which most were in the family of SCP/TAPs (sperm coating proteins/transporter associated proteins) (110 proteins). The most abundant constituents of ESP were found to be homologues of tissue inhibitors of metalloproteases family. Moreover, among the ESP proteins, homologues of vaccine candidates and immunomodulatory proteins were also found, generating more data for future validation studies.

#### 2.5.2. *Angiostrongylus cantonensis* Infections

The nematode *Angiostrongylus cantonensis* commonly resides in the pulmonary arteries of rats and thus has received the common name ‘rat lungworm’. Snails and slugs are intermediate hosts of the parasite, where larvae develop until they become infective. Consumption of raw or undercooked terrestrial snails can lead to infection of people. Ingestion of food items contaminated by excretions of intermediate hosts or by faeces of rats can also lead to infection by *A. cantonensis* [215]. Humans are considered to be accidental hosts of the parasite. The infection can lead to eosinophilic meningitis. The infection can remain clinically inapparent, whereas neurological signs can occur in 10% of infected people, characterised by accumulation of eosinophils in cerebrospinal fluid and increased pressure [15].

The protein expression profiles of the infective third- and pathogenic fifth-stage larvae (L_3_ and L_5_) of the parasite were evaluated by 2-DE and MALDI-TOF MS. In total, 33 proteins were detected in L_3_ larvae and 67 in L_5_ larvae. In L_5_ larvae, more binding and transport-related proteins were found than in L_3_, whilst in L_3_ larvae, there was a higher expression of cytoskeleton and membrane proteins. These differences can be possibly the result of the development of L_3_ in snails to L_5_ in rats; they may be relevant to finding the stage-specific proteins and biomarkers for diagnosis of the infection [216].

#### 2.5.3. *Trichinella spiralis* spp. Infections

Trichinellosis (trichinosis) is a parasitic infection of pigs, in which animals’ parasites are located primarily in the striated muscles. People become infected after consumption of raw or undercooked meat of infected pigs. In fact, in the United States of America, 58% of the cases in humans were associated with consumption of pig meat, whilst in Central Europe, over 2000 cases have been diagnosed in which the source of infection was pigs [15].

As part of the initial proteomics works on *T. spiralis*, Robinson and Connolly [217] analysed excretory-secretory products from *T. spiralis* and *T. pseudospiralis* [218] muscle larvae by means of 2-DE gels and mass spectral analysis with MALDI-TOF MS, and found mainly secreted glycoproteins to be part of the excretory-secretory products.

The same technologies were then used to the parasite–host interface at the intestinal epithelium. Cui et al. [219], after examining larval surface proteins with the scope to identify parasite molecules that might interact with intestinal epithelial cells during infection and have a role in invasion, found 15 different proteins, five of which presented catalytic and hydrolase activity. Likewise, Liu et al. [220] compared changes in the surface proteins among muscle larvae and the intestinal infective larvae after infection to identify parasite proteins activated in the infection process as the helminths enter into the intestine of the host. Many proteins (41) were common among both phases; however, 85 and 113 additional proteins were specific for the muscle larvae and intestinal infective larvae, respectively. Those in the intestines exhibited elevation in proteins related to energy and nucleic acid metabolic processes, among others.

In a further study [221] aiming to identify immunoreactive proteins recognised by anti-*Trichinella* antibodies, muscle larvae and Ad crude extracts and their excretory-secretory (E-S) products were subjected to SDS-PAGE and LC-MS/MS with serum samples from pigs challenged with *T. spiralis*. Three proteins common for both adult stage and muscle larvae, including heat shock proteins, enolase and 5′-nucleotidase, might play important role during *T. spiralis* infection and may be possible antigens-biomarkers for the early diagnosis of the infection and the development of a vaccine against the parasite.

Gondek et al. [222] used proteomics to examine changes in host serum proteins at early (13 days after infection) and late (60 days after infection) stages in pigs experimentally infected with *T. spiralis*, *T. britovi* or *T. pseudospiralis* using 2-DE gels and MALDI-TOF. Comparisons between animals infected with each of these species and uninfected controls revealed in total 27 proteins, 15 of which could be identified with mass spectrometry. These results showed that various *Trichinella* species can produce, at different phases of the host invasion, distinct and characteristic proteomes and patterns in infected pigs. These have the potential to indicate possible biomarkers of early infection.

#### 2.5.4. *Echinococcus* spp. Infections

Human echinococcosis is a zoonotic disease that occurs as the result of infection by the larval stages of parasites of the genus *Echinococcus*. Four species of *Echinococcus* are of public health concern: *Echinococcus granulosus* (which causes cystic echinococcosis, also known as hydatid disease or hydatidosis), *Echinococcus multilocularis* (which causes alveolar echinococcosis), as well as *Echinococcus vogeli* and *Echinococcus oligarthrus* (which cause polycystic echinococcosis). Carnivores harbour the mature cestodes (tapeworms) in their intestinal tract. Humans and many animal species act as intermediate hosts of the parasite and become infected by ingesting parasite eggs (more often through contaminated food or water).

Proteomics analyses (in- and off-gel protein fractionation techniques and MS/MS) of the composition of cysts of E. *granulosus* have identified a variety of proteins, of parasite or host origin, used to understand the parasite survival strategies and the parasite–host interaction mechanisms. In total, 130 proteins were found in cysts from sheep, cattle or people [223], whilst 153 proteins were detected in the hydatic fluid of *E. granulosus* cysts and 120 in the alveolar fluid of *E. multilocularis* cysts [224]. Moreover, for *E. multilocularis*, by using LC-MS/MS, 392 proteins of parasitic origin were identified in the alveolar fluid [225]. Of note, however, is that the protein composition of hydatid fluid of *E. granulosus* cysts differs according to the organ, where cysts were localised [226]. These differences are probably related to the tissue location of the cysts and the fertility status of the parasite. The identification of host proteins in hydatid fluids may indicate that the walls of the cysts are permeable, thus allowing a high protein exchange rate between host and parasite [226].

In addition, the analysis of proteins leads to the identification of molecular markers for the development of diagnostic methods and monitoring of the disease course. By means of LC-MS/MS, nine proteins found to be more abundant in patients not responding to albendazole treatment could be employed as potential biomarkers to monitor the course and outcome of the infection during treatment. Specific immunodominant epitopes of *E. granulosus* hydatid fluid change as the disease progresses [227]. The findings could explain the differences in the host response, in accord with stage and localisation of cysts and, in the future, could contribute to application of proteomics to patient care management [228].

## 3. Proteomics Applications in Antibiotic Resistance

The use of proteomics in One Health also involves the study of antibiotic-resistant bacterial isolates, which may cause zoonotic infections [229,230]. The proteomics of bacterial isolates resistant to antimicrobial agents refer to the evaluation of differential expression of proteins and post-translational modifications, with the aim to elucidate mechanisms through which bacteria develop resistance to antimicrobial agents and to evaluate novel treatments against such organisms (Table S3).

The analysis of modifications in the pattern of protein expression in response to antimicrobial agents and the classification of the differentially expressed microbial proteins leads to disclosure of specific responses that may occur after drug administration constructing the molecular networks and pathways. Detection of post-translational modifications associated with antimicrobial resistance supports the identification of potential targets for preventing the development of resistance [231]. Recent studies have shown that protein post-translational modifications can play a role in bacterial antibiotic resistance. One such type of post-translational modification, lysine acetylation, is a reversible and highly regulated modification, which has been found to be associated with antibiotic resistance [232].

Li et al. [233] have provided mechanistic insights into high-level resistance to chloramphenicol in *C. jejuni*, using integrated genomics and proteomics analyses. Two radical S-adenosylmethionine (SAM) enzymes and a differentially expressed protein were determined. The results pointed out to a new mechanism for development of resistance in *Campylobacter* strains, a methylation mechanism by an enzyme of the SAM super-family. This was based on the detection of changes in oxidative phosphorylation and ABC transporters, indicative of accumulation of energy and the increase in the import of methionine.

The proteome of a fluoroquinolone-resistant *S. enterica* serovar Typhimurium Se20 isolate (phage type DT104B), recovered after ciprofloxacin treatment, was studied compared to the proteome of a susceptible reference isolate SL1344. In the isolate recovered after antibiotic administration, three proteins related to antimicrobial resistance were detected [234]. Subsequently, ciprofloxacin-resistant and -susceptible isolates of *Salmonella* were used and tandem mass tag labelling and acetylation enrichment techniques were employed to screen for the different expression of acetylated proteins between the two isolates and for quantitative and bioinformatics analysis. In total, 631 acetylated proteins involving 1259 lysine acetylation sites were detected. Among the quantified sites, compared with the susceptible strain, the expression of lysine acetylation was upregulated for 112 sites and downregulated for 149 sites in the resistant strain. Bioinformatics analyses has indicated that the main enrichment pathways for these differentially acetylated proteins were microbial metabolic process, biosynthesis of antibiotics and bacterial chemotaxis. Among the differentially acetylated proteins, 14 proteins related to bacterial antibiotic resistance were identified (excluding metabolic and virulence-related proteins), and the lysine acetylation expression of these proteins was significantly different between the resistant and susceptible strains [232].

In a different approach, Radford et al. [235], studied an isolate of serovar Enteritidis before and after induction of resistance to ceftiofur. Proteomics comparison of the two lineages of the organism indicated alteration of specific drug-, heme-, sugar-, amino acid- and sulphate-transporters, whilst the localisation of the cell membrane stabilising protein OsmY was also modified. This redistribution was responsible for minimising the concentrations of ceftiofur in the periplasm, by decreasing facilitated import and increasing active efflux and cytosolic sequestration. Changes in specific regulators of post-translational dynamics in the derived ceftiofur-tolerant lineages decreased metabolic strain on cell walls and enhanced the stability of the periplasmic envelop.

Extensive work has been performed with bacterial isolates associated with mastitis in animals. Dairy products are usually not implicated in the dissemination of antimicrobial resistance to consumers of milk, as bacteria would be destroyed during pasteurisation. However, cell-free genetic material of staphylococcal isolates resistant to antimicrobial agents, which would not be destroyed during thermal processing of milk, might possibly be transferred to humans [236,237]. Resistance genes could be incorporated in other bacterial species (e.g., *Streptococcus* spp., *Acinetobacter* spp.) that are part of the normal bacterial flora of humans, leading to the dissemination of resistance genes. This way, staphylococcal isolates in milk can act as ‘stores’ of resistant genes and dairy products a means for their transfer [238].

Mapping of surface proteins of *S. aureus*, using isolates from disease- or carrier- cases, has provided a benchmark for strain comparison of pathogens with pathogenic characteristics and antibiotic resistance mechanism. This may be useful in defining therapeutic targets. This was carried out by Taverna et al. [239] by use of proteomics approach on extracts of lysostaphin-treated *S. aureus* to produce a reproducible and well resolved reference map of surface proteins of the organism. Liu et al. [240] applied label-free quantitative proteomics and detected 200 proteins to be differentially expressed in SIPI-8294 (an erythromycin derivative)/oxacillin-treated cells of *S. aureus*; there were responses differing to those of drugs given individually or even to that of erythromycin/oxacillin combination. The findings have indicated new therapeutic approaches for mastitis. Furthermore, methicillin-resistant isolates of *S. aureus* were studied in depth by means of proteomics (2-DE LC MS/MS and shotgun LC MS/MS) [241] for comparison and elucidation of the antimicrobial-resistance mechanisms. A higher frequency of proteins were found in resistant organisms, associated with the processes of glycolysis, protein biosynthesis, oxidation-reduction process, stress response, ATP hydrolysis-coupled proton transport and 1-carbon metabolism. This showed a change in cell physiology in all processes that fulfil an important function in maintaining normal cell function. Moreover, proteins with more specific functions, e.g., catalase and superoxide dismutase, were found in resistant isolates, indicating that these proteins can protect intraphagocytic bacteria by potentially destroying hydrogen peroxide produced by the phagocyte [242].

With regard to streptococci, Abril et al. [243], using LC-ESI MS/MS (liquid chromatography-electrospray ionisation tandem mass spectrometry) in milk samples from cows with mastitis caused by streptococci, identified 134 peptides specific to *Streptococcus* spp.—to be representing proteins that corresponded to virulence factors, toxins and anti-toxins of these bacteria—that provided resistance to antimicrobial agents associated with the production of antibiotic-related compounds, or to play a role in the resistance to toxic substances. These results could provide new targets of therapeutics for *Streptococcus* spp. isolates causing mastitis.

Moreover, the proteome of an extended-spectrum *β*-lactamase (ESBL) producing *E. coli* isolate, recovered from faeces of pigs, was studied [244]. In order to evaluate the response of this isolate to stress, an increased concentration of ciprofloxacin was applied, resulting in overexpressing hydrolase L-asparaginase. This can lead to a diverse secondary response by influencing the production of other proteins potentially involved in the mediation of development of resistance to ciprofloxacin. Differential expression of proteins linked to oxidative stress response, to DNA protection and to membrane permeability has also been reported in *E. coli* isolates resistant to enrofloxacin [245]. In relation to kanamycin-resistance of *E. coli* isolates, MS and Western blotting results revealed that outer membrane proteins TolC, Tsx and OstA were found to be upregulated and MipA, OmpA, FadL and OmpW were found to be downregulated in such isolates. Further to these findings, the expression of MipA in response to four other antibiotics (nalidixic acid, streptomycin, chloramphenicol, chlortetracycline) was also studied [246].

Proteomics analyses were also performed in *M. haemolytica* isolates to evaluate effects of chlortetracycline alone or in combination with sulfamethazine and it was observed that concurrent administration of the two antibacterial agents decreased the expression of leukotoxin, which is a primary virulence factor of *M. haemolytica* [247]. The same work also showed that administration of sub-inhibitory concentrations of chlortetracycline resulted in the expression of asl-lactate permease, ATPases with chaperone activity, ATP-binding subunit protein and other proteins involved in modifying RNA structure of the organism, which indicated a potential mechanism for the development of antibiotic resistance.

## 4. Proteomics Applications in Food Safety

Proteomics constitutes an interesting approach to integrate into food safety procedures linked to animal health (Table S4). Such aspects are of importance for monitoring zoonotic agents that may be transmitted to humans through food [248,249]. By using proteomics methodologies, one may effectively monitor the quality of production, identify potential threats of consumer health and detect possible spoilage. Proteomics within the food industry is a growing field, which is part of the recently termed ‘foodomics’ approach [250]. Foodomics aims to increase knowledge regarding food quality, food safety, food traceability and food bioactivity in order to safeguard public health by means of biomics technologies [251]. As part of food safety, proteomics can also be employed to detect proteins acting as allergens in foods [252].

Proteomics in the food industry is a growing sector and enables the integration into food production of processes that are related to animal health and concern the monitoring of pathogens with a potential for transmission to humans through food [253]. Proteomics technologies can monitor the quality of food production and identify potential threats to consumer health, e.g., the presence of enterotoxins in dairy products.

The importance of proteomics methodologies for food safety was understood early and presented already in the initial stages of the development of the technologies. Thus, Piñeiro et al. [254] proposed a variety of potential applications of proteomics for investigation of seafood products: the detection of shellfish toxins, the detection of allergens, the precise identification of the species, the evaluation of the developmental stage of the aquatic species, the characterisation of post-mortem changes in fish and crustacean muscles, the evaluation of processing conditions and the presence of contaminants in the water environment.

### 4.1. Detection of Pre-Harvest Contaminants and Post-Harvest Changes

Pre-harvest contamination of animal tissues as a consequence of environmental factors, e.g., in food of aquatic origin [255], may be identified by means of proteomics. Moreover, the same methodology can also help to study pre-slaughter stress of farm animals [256].

Post-harvest changes may also be detected by means of proteomics usage, e.g., oxidative damage in milk products [257], inappropriate maturation process in dairy products [258], untargeted changes in porcine meat [259] and technological errors in dry-cured ham [260].

Proteomics iTRAQ-based techniques have been established for comparative proteomics analysis to compare the protein profiles between a parental *Lactobacillus casei* and its amoxicillin-resistant descendent clones in order to study the adaptation of *L. casei* to amoxicillin stress; 38 proteins were found to increase, whilst 34 were found significantly decreased. The differences in the proteomic profiles between the two strains might explain the enhanced stress resistance of the adapted bacteria [261].

### 4.2. Study of Product Traits

The general objectives of proteomics studies in foods are to characterise the quality and the processing conditions of the animal products in order to ensure and predict the quality of the end-product. In that context, with regard to meat quality characteristics, tenderness [262,263], juiciness [264] and flavour and odour [265,266,267] have been studied. Identification of naturally generated small peptides derived from myofibrillar proteins (e.g., myosin light chain I, titin, actin) would promote understanding of the various proteinases affecting food properties.

Products of animal origin, in which traits have been studied with proteomics methodologies, also include eggs [268,269], cheese [270] and honeybee products [271,272]. Such studies served to ensure compliance of the products with norms related to the respective products.

### 4.3. Identification of Pathogens and Toxins

Various studies have been performed to evaluate characteristics of bacteria recovered from food of animal origin.

Cain et al. [273] noted that glycosylation (seen by applying label-based LC MS/MS) played a role in *C. jejuni* isolates from samples from slaughtered poultry. These included biofilm-formation, motility, cell stress, nutrient uptake and utilisation and chemosensing. Another application of proteomics techniques is the precise and accurate identification of bacterial strains and is used additionally in microbiological studies. A relevant example is the differentiation of *Enterococcus faecium* from other bacterial species from processed meat samples [274].

Moreover, Witt et al. [275] presented methodologies (LC-MS/MS) for the detection of *F. tularensis* in carcasses of hares. Another study in meat investigated the application of MALDI-TOF MS for the detection of *L. monocytogenes*, *S. enterica* and *E. coli* O157:H7 in red-meat samples. Species-level identification was achieved within 18 to 30 h after collection of the samples and putative biomarkers relating to the above organisms were evident within 24 h after contamination of meat with the above bacteria [276].

With regard to aquatic products, Zhu et al. [277] obtained proteomic profiles of *Vibrio parahaemolyticus* recovered from commonly consumed aquatic products (e.g., mussels) and identified candidate protein spots by using 2-DE and LC MS/MS. In total, 11 common and 28 differential extracellular proteins was found, including eight virulence-associated proteins—outer membrane channel TolC, maltoporin, elongation factor Tu, enolase, transaldolase, flagellin C, polar flagellin B/D and superoxide dismutase—as well as five antimicrobial and/or heavy metal resistance-associated ABC transporter proteins. Comparison of proteomic profiles derived from the 12 *V. parahaemolyticus* isolates also revealed five intracellular virulence-related proteins, including aldehyde-alcohol dehydrogenase, outer membrane protein A, alkyl hydroperoxide reductase C, phosphoenolpyruvate-protein phosphotransferase and phosphoglycerate kinase.

With regard to toxin detection, proteomics methodologies have been employed to detect toxins of *S. aureus*, specifically staphylococcal enterotoxin A and staphylococcal enterotoxin B, in meat, milk and derived products, [278,279,280]. In particular, the results of Andjelkovic et al. [279] indicated that the toxins could be successfully detected in milk samples and the method had an excellent sensitivity with a very small detection limit (<8 ng g^−1^), which is comparable or even lower than those achieved with other toxin detection protocols [279].

Targeted proteomics techniques were developed for the detection and quantification of three toxins in food matrices (milk and tap water): ricin (a *Ricinus communis* toxin), staphylococcal enterotoxin B and *Clostridium perfringens* epsilon toxin (ETX). At least seven peptides were targeted for each toxin (43 peptides in total) with a quadrupole-Orbitrap instrument. Quantification was performed using stable isotope-labelled toxin standards spiked before immunocapture [281]. The same researchers later developed methodologies (LC-SRM, liquid chromatography-selected reaction monitoring) for thr quantification of the following toxins in food matrices: ricin, ETX, staphylococcal enterotoxin A, staphylococcal enterotoxin B, shigatoxins from *Shigella dysenteriae* and enterohaemorragic *E. coli* strains and *C. jejuni* cytolethal distending toxin [282].

Various mycotoxins (fungal metabolites) can be toxic for humans and animals. The Fungal Secretome Database [283] can serve as an integrated platform supporting research on secretory fungal proteins. Proteomics approaches can provide a means for the identification of proteins that are involved in fungal development, interactions between the host and parasitic fungi and fungal pathogenesis [284].

Nzoughet et al. [285] have used proteomics as a tool for the characterisation of proteins in blue mussels (*Mytilus edulis*) contaminated with azaspiracid toxins (AZA), in order to study possible biomarkers. These molluscs were found to contain AZA-1, AZA-2 and AZA-3 toxins, which were identified by peptide mass fingerprinting and MS/MS analysis through nano-LC-ESI–MS/MS and MALDI-TOF/TOF/MS. This knowledge would support the development of processes for the depuration of AZA-contaminated shellfish [286].

### 4.4. Detection of Allergens

Cow milk allergy (CMA) is a common food allergy, primarily during childhood. The main cow milk allergens are caseins, *β*-lactoglobulin and *α*-lactalbumin [287]. Proteomics techniques have been used to identify such allergies and also to discover hidden allergens in food matrices, as more allergic patients react also to traces of allergens contained in complex matrix or foodstuff [288]. Moreover, further innovative applications enable the use of proteomics in ‘personalised medicine’ that may evaluate people’s unique characteristics at the molecular level [288].

Proteomics studies and analysis of allergenic proteins have been critical in determining the safety of aquatic food products [289]. The major allergen identified in fish was *β*-parvalbumin. A rapid strategy for the detection of *β*-parvalbumin was performed the use of targeted proteomics by SMIM (Selected MS/MS Ion Monitoring) [290]. In contrast, in shrimps and molluscs, tropomyosin was found to be the major allergen. Proteomics profiling of tropomyosin was performed to obtain the full amino acid sequence in a Q-TOF (Quadrupole-time of flight) instrument [291].

### 4.5. Exposure of Adulteration

Post-database processing was performed to obtain confident peptide sequence assignments with the aim to detect accurately milk adulteration, even with inclusion rates lower than 1%. Species-specific peptides from bovine *β*-lactoglobulin and *α* S1 casein were identified as suitable peptide markers of authenticity of cow milk [292].

## 5. Conclusions

The applications of proteomics in ‘One Health’ primarily include the control of zoonoses. These aimed to study the protein–protein interactions or post-translational modifications involved in the pathogenesis of the disease, as well as detecting biomarkers for early diagnosis and therapeutic targets. Further, proteomics studies of the mechanisms of adapting resistance by various bacteria to antimicrobial agents have also been performed. Finally, various proteomics applications have been employed in food safety, included at the term ‘foodomics’ and refer to an increase in available knowledge of the quality and traits of foods, the study of food-borne pathogens and the detection of possible allergens or adulterations at food matrices.

The study of interactions between genes and their environment, thus of molecules that form the ‘functional genome’, has been enhanced by the expansion of the omic technologies. Proteomics have nowadays become an important research tool for life scientists because of their use of protein characterisation and biomarker discovery.

Proteomics implements high-throughput technologies, which are constantly improving. The rapid evolution of high-throughput technologies allows the production of large-scale data on the DNA, RNA and protein levels in various tissues. Further, sophisticated computational tools can help to integrate those data sets, with the aim to enhance information. Such data are continuously employed in approaches when the complete genome has not been completely sequenced, as is the case with some farm animals.

Concurrently, technological advancements in the area of mass spectrometry, the success of the genome projects and the development and wide dissemination of bioinformatics tools have contributed to the advancement of the proteomics approaches and methodologies. In the future, sensitive analysis of several hundreds of proteins, including the low-abundant ones in complex biological samples, will be achieved. Proteomics are particularly useful and offer novel perspectives in the understanding of the pathophysiology of various infection processes and capitalise on comparative studies to provide answers.

All the above will ultimately enable the implementation of targeted proteomics in clinical laboratory settings, shedding more light on biomarker research and ultimately the promotion of the One Health concept.

## Data Availability

Not applicable.

## Supplementary Materials

The following are available online at https://www.mdpi.com/article/10.3390/proteomes9030031/s1, Table S1: Proteomics studies with a significance in the ‘One Health’ context, which were performed in animal samples, reviewed in the text of the paper, Table S2: Summary of the milestones COVID-19 research by use of proteomics methodologies, Table S3: Proteomics studies of antibiotic resistance, which were performed in animal samples, reviewed in the text of the paper, Table S4: Proteomics studies, which were performed in samples of food of animal origin, reviewed in the text of the paper.
