# Supplementary material for: Applied Proteomics in ‘One Health’
Source: Proteomes. 2021 Jun 30;9(3):31. doi: 10.3390/proteomes9030031 (PMC8293331; doi:10.3390/proteomes9030031)
Supplement: Supplementary file 1 [file proteomes-09-00031-s001.zip › proteomes-1203601-supplementary.pdf]

Applied Proteomics in 'One Health'

Eleni I. Katsarou, Charalambos Billinis, Dimitrios Galamatis, George C. Fthenakis, George Th. Tsangaris, and Angeliki I. Katsafadou

Table S1. Proteomics studies with a significance in the 'One Health' context, which were performed in animal samples, reviewed in the text of the paper.

Pathogen	Animal species	Type of sample(s)	Proteomics methodologies applied [1]	Summary of main findings	Reference
Prion protein	Sheep	Blood serum	SELDI-TOF MS	Potential biomarkers	Batxelli-Molina et al. 2010
	Sheep	Brain tissue	MALDI-TOF MS	Proteins associated with pathogenesis	Morel et al. 2007
	Sheep	Brain tissue	2-DE, LC-MS/MS	Proteins associated with pathogenesis	Petrakis et al. 2009
	Hamster	Brain tissue	2-DE, MALDI-TOF, LC-MS/MS	Proteins associated with pathogenesis	Giorgi et al. 2009
	Mouse	Brain tissue	SDS PAGE, LC-MS/MS	Potential biomarkers	Graham et al. 2011
Rabies Virus	Dog	Brain tissue	2-DE, Q-TOF MS, MS/MS	Proteins associated with pathogenesis<br>Protein networks	Thanomsridetchai et al. 2011
	Dog	Spinal cord tissue	2-DE, Q-TOF MS, MS/MS	Proteins associated with pathogenesis<br>Protein networks	Thanomsridetchai et al. 2011
	Dog	Brain tissue	2-DIGE	Potential biomarkers	Kasempimolporn et al. 2014
	Cat	Brain tissue	2-DIGE	Potential biomarkers	Kasempimolporn et al. 2014
	Mouse	Neuronal tissue	2-DE, MS	Potential biomarkers	Mehta et al. 2016
Influenza Virus	Poultry	Trachea	2-DIGE, MALDI-TOF MS/MS	Proteins associated with pathogenesis	Sun et al. 2014
	Poultry	Brain tissue	2-DE, MALDI-TOF MS/MS	Proteins associated with pathogenesis	Zou et al. 2010
M. avium subsp. *paratuberculosis*	Sheep	Blood serum	2-DE, MALDI-TOF MS	Proteins associated with pathogenesis	Hughes et al. 2008
	Sheep	Intestinal tissue	2-DE, MALDI TOF MS	Proteins associated with pathogenesis	Hughes et al. 2007
	Sheep	Blood serum	SELDI-TOF MS	Proteins associated with pathogenesis	Zhong et al. 2011
M. bovis	Cattle	Blood serum	iTRAQ, LC MS/MS	Potential biomarkers	Seth et al. 2009
	Cattle	Blood serum	iTRAQ	Potential biomarkers	Lamont et al. 2014
	Cattle	Blood serum	RP-LC-MS/MS	Proteins associated with pathogenesis	Lopez et al. 2018
B. abortus	Cattle	Blood serum	1-DE SDS PAGE, 2-DE	Proteins associated with pathogenesis<br>Potential biomarkers	Pajuaba et al. 2012
B. melitensis	Sheep	Blood serum	LC-MS	Potential biomarkers	Mavromatti 2012

B. mallei	Horse	Blood serum	2-DE, MALDI-TOF/TOF	Potential biomarkers	Dohre et al. 2017
	Horse	Blood serum	SDS PAGE	Potential biomarkers	Yazdansetad et al. 2019
C. jejuni	Sheep	Blood serum	2-DE, MALDI-TOF	Proteins associated with pathogenesis<br>Potential biomarkers	Wu et al. 2014
	Poultry	Intestinal tissue	iTRAQ, 2-DE, LC MS/MS	Proteins associated with pathogenesis<br>Potential biomarkers	Asakura et al. 2016
	Poultry	Intestinal tissue	2-DIGE, nano LC-MS/MS	Proteins associated with pathogenesis	O'Reilly et al. 2017
	Poultry	Skin	LC MS	Proteins associated with pathogenesis	Taniguchi et al. 2021
S. enterica ser. Typhimurium	Pig	Intestinal tissue	2-DIGE	Proteins associated with pathogenesis	Arce et al. 2014
	Pig	Lymph node	2-DIGE, MALDI TOF/TOF	Proteins associated with pathogenesis	Martins et al. 2012
	Pig	Intestinal tissue	iTRAQ, LC MS/MS	Proteins associated with pathogenesis	Collado-Romero et al. 2015
	Pig	Intestinal tissue	2-DE, MALDI-TOF	Proteins associated with pathogenesis	Collado-Romero et al. 2012
S. enterica ser. Enteritidis	Poultry	Blood serum	TMT, LC MS/MS	Proteins associated with pathogenesis<br>Potential biomarkers	Polansky et al. 2018
	Poultry	Liver tissue	TMT, LC MS/MS	Proteins associated with pathogenesis<br>Potential biomarkers	Polansky et al. 2018
	Poultry	Spleen tissue	LC MS/MS	Proteins associated with pathogenesis	Sekelova et al. 2017
L. copenhageni	Rats	Urine	2-DIGE, nano LC-MS/MS	Potential biomarkers	Nally et al. 2011
	Rats	Urine	1-DE SDS PAGE, 2-DE	Proteins associated with pathogenesis	Monahan et al. 2008
L interrogans	Hamsters	Kidney & liver tissue	2-DE, MALDI-TOF	Proteins associated with pathogenesis	Vieira et al. 2009
M. haemolytica	Sheep	Blood serum	2-DE, MALDI-TOF MS	Proteins associated with pathogenesis	Katsafadou et al. 2019a
	Sheep	Milk	2-DE, MALDI-TOF MS	Proteins associated with pathogenesis	Katsafadou et al. 2019a
	Sheep	Milk	2-DE, MALDI-TOF MS	Potential biomarkers	Katsafadou et al. 2019b
M. agalactiae	Sheep	Milk	2-DIGE, SDS PAGE, GeLC-MS/MS	Proteins associated with pathogenesis<br>Potential biomarkers	Addis et al. 2011
S. aureus	Sheep	Blood serum	SDS PAGE	Proteins associated with pathogenesis	Le Maréchal et al. 2009
	Cattle	Milk	CE MS, LC MS/MS	Potential biomarkers	Mansor et al. 2013
S. uberis	Sheep	Milk	2-DIGE, SDS PAGE	Proteins associated with pathogenesis<br>Potential biomarkers	Addis et al. 2013
	Sheep	Milk	LC-MS/MS	Proteins associated with pathogenesis	Pisanu et al. 2015
	Sheep	Mammary tissue	LC-MS/MS	Proteins associated with pathogenesis	Pisanu et al. 2015
L. infantum	Dog	Blood serum	RP-LC-MS/MS	Potential biomarkers	Franco-Martinez (2020)
T. spiralis	Pig	Blood serum	2-DE, MALDI-TOF	Proteins associated with pathogenesis<br>Potential biomarkers	Gondek et al. (2020)

[1] 1-DE SDS-PAGE: One-Dimensional Electrophoresis Sodium Dodecyl Sulphate–PolyAcrylamide Gel Electrophoresis; 2-DE: Two-Dimensional Electrophoresis; 2-DIGE: Two-Dimensional Difference Gel Electrophoresis; CE: Capillary Electrophoresis Mass Spectrometry; GeLC-MS/MS: Gel electrophoresis Liquid Chromatography-Mass Spectrometry/Mass Spectrometry; iTRAQ: isobaric Tag for Relative and Absolute Quantitation; LC-MS: Liquid Chromatography-Mass Spectrometry; LC-MS/MS: Liquid Chromatography-Mass Spectrometry/Mass Spectrometry; MALDI TOF: Matrix-Assisted Laser Desorption/Ionization – Time Of Flight; MALDI TOF/TOF: Matrix-Assisted Laser Desorption/Ionization – Time Of Flight/Time Of Flight; MS: Mass Spectrometry; Q-TOF MS: Quadrupole-Time Of Flight Mass Spectrometry; RP-LC-MS/MS: Reverse Phase-Liquid Chromatography-Mass Spectrometry/Mass Spectrometry; SELDI-TOF: Surface-Enhanced Laser Desorption/Ionization – Time of Flight; SDS-PAGE: Sodium Dodecyl Sulphate–PolyAcrylamide Gel Electrophoresis; TMT: Tandem Mass Tags.

References

Addis, M.F.; Pisanu, S.; Ghisaura, S.; Pagnozzi, D.; Marogna, G.; Tanca, A.; Biosa, G.; Cacciotto, C.; Alberti, A.; Pittau, M.; Roggio, T.; Uzzau, S. Proteomics and pathway analyses of the milk fat globule in sheep naturally infected by Mycoplasma agalactiae provide indications of the in vivo response of the mammary epithelium to bacterial infection. *Infect. Immun.* **2011**, *79*, 3833–3845.

Arce, C.; Lucena, C.; Moreno, A.; Garrido, J.J. Proteomic analysis of intestinal mucosa responses to *Salmonella* enterica serovar Typhimurium in naturally infected pig. *Comp. Immunol. Microbiol. Infect. Dis.* **2014**, *37*, 59–67.

Asakura, H.; Kawamoto, K.; Murakami, S.; Tachibana, M.; Kurazono, H.; Makino, S.; Yamamoto, S.; Igimi, S. *Ex vivo* proteomics of *Campylobacter jejuni* 81-176 reveal that FabG affects fatty acid composition to alter bacterial growth fitness in the chicken gut. *Res. Microbiol.* **2016,** 167, 63–71.

Batxelli-Molina, I.; Salvetat, N.; Andréoletti, O.; Guerrier, L.; Vicat, G.; Molina, F.; Mourton-Gilles, C. Ovine serum biomarkers of early and late phase scrapie. *Vet. Res.* **2010**, *6*, 49.

Collado-Romero, M.; Aguilar, C.; Arce, C.; Lucena, C.; Codrea, M. C.; Morera, L.; Bendixen, E.; Moreno, Á.; Garrido, J.J. Quantitative proteomics and bioinformatic analysis provide new insight into the dynamic response of porcine intestine to *Salmonella* Typhimurium. *Fr. Cell. Infect. Microbiol.* **2015**, *5*, 64.

Dohre, S.K.; Kamthan, A.; Singh, S.; Alam, S.I.; Kumar, S. Identification of a new diagnostic antigen for glanders using immunoproteome analysis. *Comp. Immunol. Microbiol. Infect. Dis*. **2017**, *53*, 26–32.

Franco-Martínez, L.; Villar, M.; Tvarijonaviciute, A.; Escribano, D.; Bernal, L. J.; Cerón, J.J.; Thomas, M.; Mateos-Hernández, L.; Tecles, F.; de la Fuente, J.; López, M.C.; Martínez-Subiela, S. Serum proteome of dogs at subclinical and clinical onset of canine leishmaniosis. *Transbound. Emerg. Dis.* **2020**, *67*, 318–327.

Giorgi, A.; Di Francesco, L.; Principe, S.; Mignogna, G.; Sennels, L.; Mancone, C.; Alonzi, T.; Sbriccoli, M.; De Pascalis, A.; Rappsilber, J.; Cardone, F.; Pocchiari, M.; Maras, B.; Schininà, M. E. Proteomic profiling of PrP27-30-enriched preparations extracted from the brain of hamsters with experimental scrapie. *Proteomics* **2009**, *9*, 3802–3814.

Gondek, M.; Herosimczyk, A.; Knysz, P.; Ożgo, M.; Lepczyński, A.; Szkucik, K. Comparative Proteomic Analysis of serum from pigs experimentally infected with *Trichinella spiralis*, *Trichinella britovi*, and *Trichinella pseudospiralis. Pathogens* **2020**, *9*, 55.

Graham, J.F.; Kurian, D.; Agarwal, S.; Toovey, L.; Hunt, L.; Kirby, L.; Pinheiro, T.J.; Banner, S.J.; Gill, A.C. Na+/K+-ATPase is present in scrapie-associated fibrils, modulates *PrP* misfolding *in vitro* and links *PrP* function and dysfunction. *PloS One* **2011**, *6*, e26813.

Hughes, V.; Smith, S.; Garcia-Sanchez, A.; Sales, J.; Stevenson, K. Proteomic comparison of *Mycobacterium avium* subspecies *paratuberculosis* grown in vitro and isolated from clinical cases of ovine paratuberculosis. *Microbiology* **2007**, *153*, 196-205.

Hughes, V.; Bannantine, J.P.; Denham, S.; Smith, S.; Garcia-Sanchez, A.; Sales, J.; Paustian, M.L.; Mclean, K.; Stevenson, K. Immunogenicity of proteome-determined *Mycobacterium avium* subsp. *paratuberculosis*-specific proteins in sheep with paratuberculosis. *Clin. Vaccine Immunol.* **2008**, *15*, 1824–1833.

Kasempimolporn, S.; Lumlertdacha, B.; Chulasugandha, P.; Boonchang, S.; Sitprija, V. Alterations in brain cerebral cortex proteome of rabies-infected cat. *Southeast Asian J. Trop. Med. Public Health* **2014**, *45*, 808–815.

Katsafadou, A.I.; Tsangaris, G.T.; Anagnostopoulos, A.K.; Billinis, C.; Barbagianni, M.S.; Vasileiou, N.G.C.; Spanos, S.A.; Mavrogianni, V.S.; Fthenakis, G.C. Differential quantitative proteomics study of experimental *Mannheimia haemolytica* mastitis in sheep. *J. Proteom.* **2019**, *205*, 103393.

Katsafadou, A.I.; Tsangaris, G.T.; Vasileiou, N.G.C.; Ioannidi, K.S.; Anagnostopoulos, A.K.; Billinis, C.; Fragkou, I.A.; Papadopoulos, I.; Mavrogianni, V.S; Michael, C.K.; Addis, F.; Fthenakis, G.C. Detection of cathelicidin-1 in the milk as an early indicator of mastitis in ewes. *Pathogens* **2019**, *8*, 270.

Lamont, E.A.; Janagama, H.K.; Ribeiro-Lima, J.; Vulchanova, L.; Seth, M.; Yang, M.; Kurmi, K.; Waters, W.R.; Thacker, T.; Sreevatsan, S. Circulating *Mycobacterium bovis* peptides and host response proteins as biomarkers for unambiguous detection of subclinical infection. *J. Clin. Microbiol.* **2014**, *52*, 536–543.

Le Maréchal, C.; Jan, G.; Even, S.; McCulloch, J. A.; Azevedo, V.; Thiéry, R.; Vautor, E.; Le Loir, Y. Development of serological proteome analysis of mastitis by *Staphylococcus aureus* in ewes. *J. Microbiol. Methods* **2009**, *79*, 131–136

Lopez, V.; van der Heijden, E.; Villar, M.; Michel, A.; Alberdi, P.; Gortázar, C.; Rutten, V.; de la Fuente, J. Comparative proteomics identified immune response proteins involved in response to vaccination with heat-inactivated *Mycobacterium bovis* and mycobacterial challenge in cattle. *Vet. Immunol. Immunopathol*. **2018**, *206*, 54–64

Mansor, R.; Mullen, W.; Albalat, A.; Zerefos, P.; Mischak, H.; Barrett, D.C.; Biggs, A.; Eckersall, P.D. A peptidomic approach to biomarker discovery for bovine mastitis. *J. Proteom.* **2013**, *85*, 89–98

Martins, R.P.; Collado-Romero, M.; Martínez-Gomáriz, M.; Carvajal, A.; Gil, C.; Lucena, C.; Moreno, A.; Garrido, J.J. Proteomic analysis of porcine mesenteric lymph-nodes after *Salmonella typhimurium* infection. *J. Proteom.* **2012**, *75*, 4457–4470.

Mavromati J (2012). Brucellosis and proteomics: an approach in Albania. In *Farm animal proteomics 2012*, Proceedings of the 3rd Managing Committee Meeting and 2nd Meeting of Working Groups 1, 2 & 3 of COST Action FA1002, Algarve, Portugal, 27 March 2012; Rodrigues P, Eckersall D, de Almeida A, Eds.; Wageningen Academic Publishers: Wageningen, The Netherlands, 2012, pp. 80-83.

Mehta, S.; Sreenivasamurthy, S.; Banerjee, S.; Mukherjee, S.; Prasad, K.; Chowdhary, A. 2016. Pathway analysis of proteomics profiles in rabies infection: towards future biomarkers? *Omics J. Integrativ. Biol.* **2016**, *20*, 97-109.

Monahan, A.M.; Callanan, J J.; Nally, J.E. Proteomic analysis of *Leptospira interrogans* shed in urine of chronically infected hosts. *Infect. Immun.* **2008**, *76*, 4952–4958.

Morel, N.; Andreoletti, O.; Grassi, J.; Clement, G. Absolute and relative quantification of sheep brain prion protein (*PrP*) allelic variants by matrix-assisted laser desorption/ionisation time-of-flight mass spectrometry. *Rapid Comm. Mass Spectrom.* **2007**, *21*, 4093-4100.

Nally, J.E.; Monahan, A.M.; Miller, I.S.; Bonilla-Santiago, R.; Souda, P.; Whitelegge, J.P. Comparative proteomic analysis of differentially expressed proteins in the urine of reservoir hosts of leptospirosis. *PloS One* **2011**, *6*, e26046.

O'Reilly, E.L.; Burchmore, R.J.; Sparks, N.H.; Eckersall, P.D. The effect of microbial challenge on the intestinal proteome of broiler chickens. *Proteome Sci*. **2017**, *15*, 10.

Pajuaba, A.C.; Silva, D.A.; Almeida, K.C.; Cunha-Junior, J.P.; Pirovani, C.P.; Camillo, L.R.; Mineo, J.R. Immunoproteomics of *Brucella abortus* reveals differential antibody profiles between S19-vaccinated and naturally infected cattle. *Proteomics* **2012**, *12*, 820–831.

Petrakis, S.; Malinowska, A.; Dadlez, M.; Sklaviadis, T. Identification of proteins co-purifying with scrapie infectivity. *J. Proteom.* **2009**, *72*, 690–694.

Pisanu, S.; Cubeddu, T.; Pagnozzi, D.; Rocca, S.; Cacciotto, C.; Alberti, A.; Marogna, G.; Uzzau, S.; Addis, M. F. Neutrophil extracellular traps in sheep mastitis. *Vet. Res.* **2015**, *46*, 59.

Polansky, O.; Seidlerova, Z.; Faldynova, M.; Sisak, F.; Rychlik, I. Protein expression in the liver and blood serum in chickens in response to *Salmonella* Enteritidis infection. *Vet. Immunol. Immunopathol.* **2018**, *205*, 10–16.

Sekelova, Z.; Polansky, O.; Stepanova, H.; Fedr, R.; Faldynova, M.; Rychlik, I.; Vlasatikova, L. Different roles of CD4, CD8 and γδ T-lymphocytes in naive and vaccinated chickens during *Salmonella enteritidis* infection. *Proteomics* **2017**, *17*, 1700073.

Seth, M.; Lamont, E.A.; Janagama, H.K.; Widdel, A.; Vulchanova, L.; Stabel, J.R.; Waters, W.R.; Palmer, M.V.; Sreevatsan, S. Biomarker discovery in subclinical mycobacterial infections of cattle. *PloS One* **2009**, *4*, e5478.

Sun, J.F.; Han, Z.X.; Shao, Y.H.; Cao, Z.Z.; Kong, X.G.; Liu, S.W. Comparative proteome analysis of tracheal tissues in response to infectious bronchitis coronavirus, Newcastle disease virus, and avian influenza virus H9 subtype virus infection. *Proteomics* **2014**, *14*, 1403-1423.

Taniguchi, T.; Ohki, M.; Urata, A.; Ohshiro, S.; Tarigan, E.; Kiatsomphob, S.; Vetchapitak, T; Sato, H.; Misawa, N. Detection and identification of adhesins involved in adhesion of *Campylobacter jejuni* to chicken skin. *Int. J. Food Microbiol*. **2021**, *337*, 108929.

Thanomsridetchai, N.; Singhto, N.; Tepsumethanon, V.; Shuangshoti, S.; Wacharapluesadee, S.; Sinchaikul, S.; Chen, S. T.; Hemachudha, T.; Thongboonkerd, V. Comprehensive proteome analysis of hippocampus, brainstem, and spinal cord from paralytic and furious dogs naturally infected with rabies. *J. Proteome Res.* **2011**, *10*, 4911–4924.

Vieira, M.L.; Pimenta, D.C.; de Morais, Z.M.; Vasconcellos, S.A.; Nascimento, A.L. Proteome analysis of *Leptospira interrogans* virulent strain. *Open Microbiol. J.* **2009**, *3*, 69–74.

Wu, Z.; Sahin, O.; Wang, F.; Zhang, Q. Proteomic identification of immunodominant membrane-related antigens in Campylobacter jejuni associated with sheep abortion. *J. Proteom.* **2014**, 99, 111–122.

Yazdansetad, S.; Mosavari, N.; Tadayon, K.; Mehregan, I. Development of an immunoblotting assay for serodiagnosis of *Burkholderia mallei* infection: the whole-cell proteome-based paradigm. *Iran J. Microbiol.* **2019**, *11*, 232–238.

Zhong, L.; Taylor, D.; Begg, D.J.; Whittington, R.J. Biomarker discovery for ovine paratuberculosis (Johne's disease) by proteomic serum profiling. *Comp. Immunol. Microbiol. Infect. Dis.* **2011**, *34*, 315–326.

Zou, W.; Ke, J.; Zhang, A.; Zhou, M.; Liao, Y.; Zhu, J.; Zhou, H.; Tu, J.; Chen, H.; Jin, M. Proteomics analysis of differential expression of chicken brain tissue proteins in response to the neurovirulent H5N1 avian influenza virus infection. *J. Proteome Res.* **2010**, *9*, 3789–3798

Table S2. Summary of the milestones COVID-19 research by use of proteomics methodologies.

Date	Proteomics methodologies applied [1]	Summary of main findings	Reference
24 Mar. 2020	Bioinformatics methodologies (protein sequence alignment of the full-length coronavirus spike proteins)	Assembly of draft genome of the causal virus, with alignment of the spike surface glycoprotein receptor binding domain and final results indicating the pangolin as a missing link in the transmission of the virus to humans	Zhang et al. 2020
30 Apr. 2020	nano LC-MS/MS	Interactomics study with viral proteins individually produced in host cells	Gordon, Jang et al. 2020
14 May. 2020	LC-MS/MS	Identification of two protein clusters in the blood of COVID-19 patients (first: proteins downregulated during infection, mainly involved in cholesterol metabolism; secong: proteins upregulated after infection, which included RNA-modifying proteins), which could be used for potential therapeutic interventions	Bojkova et al. 2020
27 May. 2020	nano LC-MS/MS	Presentation of 14 peptides from the nucleoprotein, spike and membrane glycoprotein structural proteins for development of diagnostic techniques	Gouveia et al. 2020
22 Jun. 2020	nano-HPLC-MS/MS	Development of a technique for detection of proteins of the virus from gargle solution samples of COVID-19 patients	Ihling et al. 2020
23 Jun. 2020	Bioinformatics methodologies (727 unique protein sequences categorized by each of the 10 SARS-CoV-2 genes)	Reporting of peptides of the causal virus for potential use in multi-epitope vaccines	Yarmarkovich et al. 2020
9 Jul. 2020	LC-MS/MS	Identification of changes in the blood serum of COVID-19 patients, showing dysregulation of macrophages, platelet degranulation, complement system pathways, and massive metabolic suppression, with protein and metabolite changes, for use in selection of potential biomarkers	Shen et al. 2020
17 Jul. 2020	LC-MS/MS	Description of changes in the expression of three structural proteins and three non-structural proteins of the causal virus, consequently to *in vitro* infection of cell culture	Grenga et al. 2020
21 Jul. 2021	TMT-labeled LC-MS/MS	Presence of increased IL-6 and IL-8 in PBMCs from patients with severe disease and the functional annotation of differentially expressed proteins, implicating pathways involved in neutrophil activation, T cell receptor signaling and the coagulation cascade	Li, Guo et al. 2021
25 Jul. 2020	Bioiformatics methodologies (analysis of data from proteomic platforms)	Presentation of novel information on the presence of intrinsic disorder at the cleavage sites of replicase polyprotein 1ab of SARS CoVs	Giri et al. 2021
27 Jul. 2020	LC-MS	Identification of 27 potential biomarkers for prediction of the severity of COVID-19	Messner et al. 2020
6 Aug. 2020	nano LC-MS/MS	Rewiring of phosphorylation on host and viral proteins; promotion of casein kinase II (CK2) and p38 MAPK activation, production of diverse cytokines and shutdown of mitotic kinases by the causal virus, leading in cell cycle arrest; stimulation of marked induction of CK2-containing filopodial protrusions possessing budding viral particles; identification of drugs and compounds for potential therapeutic use	Bouhaddou et al. 2020

14 Aug. 2020	nano HPLC-MS/MS	Identification of dysregulation in various coagulation factors in COVID-19 patients, accompanied by increased levels of an-ti-fibrinolytic components, among which various serine protease inhibitors	D'Alessandro et al. 2020
10 Sep. 2020	Bioinformatics methodologies (analysis of data from proteomic platforms)	Elucidation of the pathophysiology of the 'cytokine storm', with the finding of a dominant hyperinflammatory milieu in the circulation and vascular endothelial damage markers in patients	Sims et al. 2021
10 Sep. 2020	LC-MS/MS	Identification of peptides significantly associated with SARS-CoV-2 infection and development of a multi-marker urinary peptide-based test, for enabling potential prognosis of outcomes in COVID-19 patients	Wendt et al. 2020
1 Oct. 2020	LC-MS/MS	Identification of viral protein phosphorylation, phosphorylation-driven host cell signaling changes and activation of growth factor receptor (GFR) signaling and downstream pathways are activated; description of host cell signaling events upon infection and identification of GFR signaling as a central pathway essential for replication of the causal virus	Klann et al. 2020
15 Oct. 2020	nano LC-MS/MS	Identification of differentially expressed proteins involved in cellular metabolism, blood coagulation, immune response, angiogenesis and cell microenvironment regulation, which outlined the molecular pathological features of the lung response during the infection	Leng et al. 2020
20 Oct. 2020	LC-MS/MS	Validation of transcriptome findings from colonic samples and identification of key proteins (e.g., SARS-CoV-2 entry-associated protease cathepsins B and L, inflammatory response modulator S100A8/A9) with increased expression in patients with severe disease	Wu et al. 2020
24 Oct. 2020	LC-MS/MS	Identification of 358 differentially expressed proteins in bronchoalveolar lavage fluid samples from patients with COVID-19, identified as biomarkers or therapeutic candidates for the disease	Zeng et al. 2020
17 Nov. 2020	LC-MS/MS	Identification of 11 proteins as biomarkers and a set of biomarker combinations, validated by an independent cohort for prediction of the outcomes of the disease, all these mediating variouse pathophysiological pathways (e.g., immune or inflammatory responses, platelet degranulation and coagulation, metabolism)	Shu et al. 2020
2 Dec. 2020	LC-MS/MS	Profiling of the virus-host protein-protein interactions of two hCoV nonstructural proteins (nsps) critical for viral replication	Davies et al. 2020
3 Dec. 2020	LC-MS/MS	Development and validation of an assay for detection of nucleoprotein peptides of the virus nasopharyngeal and oropharyngeal swabs	Cardozo et al. 2020
4 Dec. 2020	LC-MS/MS	Comparative viral-human protein-protein interaction and viral protein localization analyses for SARS-CoV-2, SARS-CoV-1 and MERS-CoV viruses and functional genetic screening for identification of host factors that functionally impinge on viral proliferation	Gordon, Hiatt et al. 2020
21 Dec. 2020	LC-MS/MS	Identification of 104 human proteins directly and specifically binding to SARS-CoV-2 RNAs in infected cells	Schmidt et al. 2020

29 Dec. 2020	LC-MS/MS	Identification of 91 differentially expressed proteins in plasma, depending on the severity of the disease and of 76 proteins as prognostic biomarker candidates, elucidation of the role of neutrophil activation, complement activation, platelet function and T cell suppression	Park et al. 2020
9 Jan. 2021	LC-MS-MS	Upregulation of cathepsin L1 being upregulated in lungs, identification of factors of dysregulation in hypoxia, angiogenesis, blood coagulation and fibrosis in multiple organs and presentation of evidence for testicular injuries in Covid-19 patients	Nie et al. 2021
26 Jan. 2021	LC-MS/MS	Development of a mass spectrometry assay for proteomics evaluation for SARS-CoV-2 in nasopharyngeal swab samples using common laboratory reagents	Saadi et al. 2021
8 Feb. 2021	LC-MS/MS	Identification of 13 viral proteins from nasopharyngeal swabs, indicating modulation in immune response, especially involving neutrophil and IL12-mediated signaling	Tushir et al. 2021
2 Apr. 2021	LC-MS/MS	Identification of a large dataset of SARS-CoV-2 derived peptide MS/MS spectra, specifically of two datasets with 39,650 peptides	Li et al. 2021
12 Apr. 2021	LC-MS/MS	Definition of the interactomes of SARS-CoV-2 and SARS-CoV viruses and their potential effects on the influence on the transcriptome, proteome, ubiquitinome and phosphoproteome of lung-derived human cell line	Stukalov et al. 2021
27 Apr. 2021	LC-MS/MS	Identification of dysregulation in pathways associated with peptidase activity, regulated exocytosis, blood coagulation, complement activation, leucocyte activation involved in immune response and response to glucocorticoid biological processes in severe cases of SARS-CoV-2 infection and identification of potential biomarkers for severity of the disease	Suvarna et al. 2021
3 May 2021	LC-MS	Development of an assay for diagnosis of COVID-19, based on proteolytically digested proteins of the causal virus	Van Puyvelde et al. 2021
4 May. 2021	LC-MS/MS	Identification of two potential prognostic biomarkers, involved in biological processes associated with humoral immune response, interferon signalling, acute phase response, lipid metabolism and platelet degranulation	Lee et al. 2021
20 May. 2021	LC-MS/MS	Evaluation of the heterogeneity of purified SARS-CoV-2 virus after culturing on cell line and identification of changes translated at the protein level	Gallais et al. 2021

[1] HPLC-MS/MS: High Pressure Liquid Chromatography-Mass Spectrometry/Mass Spectrometry; LC-MS: Liquid Chromatography-Mass Spectrometry; LC-MS/MS: Liquid Chromatography-Mass Spectrometry/Mass Spectrometry; TMT: Tandem Mass Tags.

References

Bojkova, D.; Klann, K.; Koch, B.; Widera, M.; Krause D.; Ciesek, S.; Cinatl, J.; Münch, C. Proteomics of SARS-CoV-2-infected host cells reveals therapy targets. *Nature* **2020**, *583*, 469–472.

Bouhaddou, M.; Memon, D.; Meyer, B.; White, K.M.; Rezelj, V.V.; Marrero, M.C.; Polacco, B.J.; Melnyk, J.E.; Ulferts, S.; Kaake, R.M. et al. The global phosphorylation landscape of SARS-CoV-2 infection. *Cell* **2020**, *182*, 685–712.e.19.

Cardozo, K.H.M.; Lebkuchen, A.; Okai, G.G.; Schuch, R.A.; Viana, L.G.; Olive, A.N.; Lazari, C.D.; Fraga, A.M.; Granato, C.F.H.; Pintao, M.C.T. et al. Establishing a mass spectrometry-based system for rapid detection of SARS-CoV-2 in large clinical sample cohorts. *Nat. Comm.* **2020**, *11*, 6201.

D'Alessandro, A.; Thomas, T.; Dzieciatkowska, M.; Hill, R.C.; Francis, R.O.; Hudson, K.E.; Zimring, J.C.; Hod, E.A.; Spitalnik, S.L.; Hansen, K.C. Serum proteomics in COVID-19 patients: altered coagulation and complement status as a function of IL-6 level. *J. Proteome Res.* **2020**, *19*, 4417–4427.

Davies, J.P.; Almasy, K.M.; McDonald, E.F.; Plate, L. 2020. Comparative multiplexed interactomics of SARS-CoV-2 and homologous coronavirus nonstructural proteins identifies unique and shared host-cell dependencies. *ACS Inf. Dis.* **2020**, *6*, 3174-3189.

Gallais, F.; Pible, O.; Gaillard, J.C.; Debroas, S.; Batina, H.; Ruat, S.; Sandron, F.; Delafoy, D.; Gerber, Z.; Olaso, R. et al. Heterogeneity of SARS-CoV-2 virus produced in cell culture revealed by shotgun proteomics and supported by genome sequencing. *Anal. Bioanal. Chem.* **2021**, in press. doi: 10.1007/s00216-021-03401-9.

Giri, R.; Bhardwaj, T.; Shegane, M.; Gehi, B.R.; Kumar, P.; Gadhave, K.; Oldfield, C.J.; Uversky, V.N. Understanding COVID-19 via comparative analysis of dark proteomes of SARS-CoV-2, human SARS and bat SARS-like coronaviruses. Cell. Mol. Life Sci. 2021, 78, 1655-1688.

Gordon, D.E.; Hiatt, J.; Bouhaddou, M.; Rezelj, V.V.; Ulferts, S.; Braberg, H.; Jureka, A.S.; Obernier, K.; Guo, J.Z.; Batra, J. et al. Comparative host-coronavirus protein interaction networks reveal pan-viral disease mechanisms. *Science* **2020**, *370*, 1181.

Gordon, D.E.; Jang, G.M.; Bouhaddou, M.; Xu, J.; Obernier, K.; White, K.M.; O'Meara, M.J.; Rezelj, V.V.; Guo, J.Z.; Swaney, D.L.; Tummino, T.A. et al. A SARS-CoV-2 protein interaction map reveals targets for drug repurposing. *Nature* **2020**, *583*, 459–468.

Gouveia, D.; Grenga, L.; Gaillard, J.C.; Gallais, F.; Bellanger, L.; Pible, O.; Armengaud, J. Shortlisting SARS-CoV-2 peptides for targeted studies from experimental data-dependent acquisition tandem mass spectrometry data. *Proteomics* **2020**, *20*, 2000107.

Grenga, L.; Gallais, F.; Pible, O.; Gaillard, J.C.; Gouveia, D.; Batina, H.; Bazaline, N.; Ruat, S.; Culotta, K.; Miotello, G. et al. Shotgun proteomics analysis of SARS-CoV-2-infected cells and how it can optimize whole viral particle antigen production for vaccines. *Emerg. Microb. Inf.* **2020**, *9*, 1712-1721.

Ihling, C.; Tanzler, D.; Hagemann, S.; Kehlen, A.; Huttelmaier, S.; Arlt, C.; Sinz, A. Mass spectrometric Identification of SARS-CoV-2 proteins from gargle solution samples of COVID-19 patients. *J. Proteom. Res.* **2020**, *19*, 4389-4392.

Klann, K.; Bojkova, D.; Tascher, G.; Ciesek, S.; Munch, C.; Cinatl, J. Growth factor receptor signaling inhibition prevents SARS-CoV-2 replication. *Mol. Cell* **2020**, *80*, 164–174.

Lee, J.S.; Han, D.; Kim, S.Y.; Hong, K.H.; Jang, M.J.; Kim, M.J.; Kim, Y.G.; Park, J.H.; Cho, S.I.; Park, W.B. et al. Longitudinal proteomic profiling provides insights into host response and proteome dynamics in COVID-19 progression. *Proteomics* **2021**, doi: 10.1002/pmic.202000278.

Leng, L.; Cao, R.Y.; Ma, J.; Mou, D.L.; Zhu, Y.P.; Li, W.; Lv, L.Y.; Gao, D.Q.; Zhang, S.K.; Gong, F. et al. Pathological features of COVID-19-associated lung injury: a preliminary proteomics report based on clinical samples. *Signal Transduct. Targ. Ther.* **2020**, *5*, 240.

Li, C.; Revote, J.; Ramarathinam, S.H.; Chung, S.Z.; Croft, N.P.; Scull, K.E.; Huang, Z.Y.; Ayala, R.; Braun, A.; Mifsud, N.A. et al. Resourcing, annotating, and analysing synthetic peptides of SARS-CoV-2 for immunopeptidomics and other immunological studies. *Proteomics* **2021**, in press. doi: 10.1002/pmic.202100036.

Li, J.; Guo, M.; Tian, X.; Wang, X.; Yang, X.; Wu, P.; Liu, C.; Xiao, Z.; Qu, Y.; Yin, Y. et al. Virus–host interactome and proteomic survey reveal potential virulence factors influencing SARS-CoV-2 pathogenesis. *Med (N.Y.)* **2021**, 2, 99–112.

Messner, C.B.; Demichev, V.; Wendisch, D.; Michalick, L.; White, M.; Freiwald, A.; Textoris-Taube, K.; Vernardis, S.I.; Egger, A.S.; Kreidl, M. et al. Ultra-high-throughput clinical proteomics reveals classifiers of COVID-19 infection. *Cell Syst.* **2020**, *11*, 11-24.e4.

Nie, X.; Qian, L.J.; Sun, R.; Huang, B.; Dong, X.C.; Xiao, Q.; Zhang, Q.S.; Lu, T.; Yue, L.; Chen, S. et al. Multi-organ proteomic landscape of COVID-19 autopsies. *Cell* **2021**, *184*, 775-761.e.14.

Park, J.; Kim, H.; Kim, S.Y.; Kim, Y.; Lee, J.S.; Dan, K.; Seong, M.W.; Han, D. In-depth blood proteome profiling analysis revealed distinct functional characteristics of plasma proteins between severe and non-severe COVID-19 patients. *Scient. Rep.* **2020**, *10*, 22418.

Saadi, J.; Oueslati, S.; Bellanger, L.; Gallais, F.; Dortet, L.; Roque-Afonso, A.M.; Junot, C.; Naas, T.; Fenaille, F.; Beche, F. Quantitative assessment of SARS-CoV-2 virus in nasopharyngeal swabs stored in transport medium by a straightforward LC-MS/MS assay targeting nucleocapsid, membrane, and spike proteins. *J. Proteome Res.* **2021**, 20, 1434–1443.

Schmidt, N.; Lareau, C.A.; Keshishian, H.; Ganskih, S.; Schneider, C.; Hennig, T.; Melanson, R.; Werner, S.; Wei, Y.J.; Zimmer, M. et al. The SARS-CoV-2 RNA-protein interactome in infected human cells. *Nat. Microbiol.* **2021**, *6*, 339-353.

Shen, B.; Yi, X.; Sun, Y.T.; Bi, X.J.; Du, J.P.; Zhang, C.; Quan, S.; Zhang, F.F.; Sun, R.; Qian, L.J. et al. Proteomic and metabolomic characterization of COVID-19 patient sera. *Cell* **2020**, *182*, 59-64.

Shu, T.; Ning, W.S.; Wu, D.; Xu, J.Q.; Han, Q.Q.; Huang, M.H.; Zou, X.J.; Yang, Q.Y.; Yuan, Y.; Bie, Y.Y. et al. Plasma proteomics identify biomarkers and pathogenesis of COVID-19. *Immunity* **2020**, *53*, 1108-1122.

Sims, J.T.; Krishnan, V.; Chang, C.Y.; Engle, S.M.; Casalini, G.; Rodgers, G.H.; Bivi, N.; Nickoloff, B.J.; Konrad, R.J.; de Bono, S. et al. Characterization of the cytokine storm reflects hyperinflammatory endothelial dysfunction in COVID-19. *J. Allerg. Clin. Immunol.* **2021**, *147*, 107-111.

Stukalov, A.; Girault, V.; Grass, V.; Karayel, O.; Bergant, V.; Urban, C.; Haas, D.A.; Huang, Y.; Oubraham, L.; Wang, A. et al. Multilevel proteomics reveals host perturbations by SARS-CoV-2 and SARS-CoV. *Nature* **2021**, *594*, 246-252.

Suvarna, K.; Biswas, D.; Pai, M.G.J; Acharjee, A.; Bankar, R.; Palanivel, V.; Salkar, A.; Verma, A.; Mukherjee, A.; Choudhury, M. et al. Proteomics and machine learning approaches reveal a set of prognostic markers for COVID-19 severity with drug repurposing potential. *Fr. Physiol.* **2021**, *12*, 652799.

Tushir, S.; Kamanna, S.; Nath, S.S.; Bhat, A.; Rose, S.; Aithal, A.R.; Tatu, U. Proteo-genomic analysis of SARS-CoV-2: a clinical landscape of single-nucleotide polymorphisms, COVID-19 proteome, and host responses. *J. Prot. Res.* **2021**, 20, 1591-1601.

Van Puyvelde, B.; Van Uytfanghe, K.; Tytgat, O.; Van Oudenhove, L.; Gabriels, R.; Bouwmeester, R.; Daled, S.; Van Den Bossche, T.; Ramasamy, P.; Verhelst, S. et al. Cov-MS: a community-based template assay for mass-spectrometry-based protein detection in SARS-CoV-2 patients. *J. Am. Chem. Soc.* **2021**, in press. doi: /10.1021/jacsau.1c00048.

Wendt, R.; Kalbitz, S.; Lubbert, C.; Kellner, N.; Macholz, M.; Schroth, S.; Ermisch, J.; Latosisnka, A.; Arnold, B.; Mischak, H. et al. Urinary peptides significantly associate with COVID-19 severity: pilot proof-of-principle data and design of a multicentric diagnostic study. *Proteomics* **2020**, *20*, 2000202.

Wu, M.; Chen, Y.B.; Xia, H.; Wang, C.L.; Tan, C.Y.; Cai, X.H.; Liu, Y.F.; Ji, F.H.; Xiong, P.; Liu, R. et al. Transcriptional and proteomic insights into the host response in fatal COVID-19 cases. *Proc. Nat. Acad. Sci. USA* **2020**, *117*, 28336-28343.

Yarmarkovich, M.; Warrington, J.M.; Farrel, A.; Maris, J.M. Identification of SARS-CoV-2 vaccine epitopes predicted to induce long-term population-scale immunity. *Cell Rep. Med.* **2020**, *1*, 100036.

Zeng, H.L.; Chen, D.; Yan, J.J.; Yang, Q.; Han, Q.Q.; Li, S.S.; Cheng, L.M. Proteomic characteristics of bronchoalveolar lavage fluid in critical COVID-19 patients. *FEBS J.* **2021**, in press. doi: 10.1111/febs.15609.

Zhang, C.; Zheng, W.; Huang, X.; Bell, E.W.; Zhou, X.; Zhang, Y. Protein structure and sequence reanalysis of 2019-nCoV genome refutes snakes as its intermediate host and the unique similarity between its spike protein insertions and HIV-1. *J. Proteome Res.* **2020**, *19*, 1351–1360.

Table S3. Proteomics studies of antibiotic resistance, which were performed in animal samples, reviewed in the text of the paper.

Bacterial pathogens	Proteomics methodologies applied [1]	Summary of main findings	Reference
Campylobacter jejuni	LC-MS/MS	New mechanism regarding rRNA methylation by a radical SAM super-family enzyme, leading to resistance to chroramphenicol	Li et al. 2017
Salmonella enterica serovar *Enteritidis*	2D-DIGE, MALDI-TOF MS	Changes of specific regulators of post-translational dynamics in resistant lineages decreased metabolic strain on cell walls and enhanced the stability of the periplasmic envelop – resistance to ceftiofur	Radford et al. 2018
Salmonella enterica serovar *Typhimurium*	1-DE SDS-PAGE, 2-DIGE, MALDI TOF/TOF MS	Elucidation of mechanisms of mechansisms of response to triclosan	Condell et al. 2012
Salmonella enterica serovar *Typhimurium*	2-DE, MALDI-TOF MS	Detection of three proteins related to antimicrobial resistance to fluoroquinolones	Correia et al. 2014
Salmonella enterica serovar *Typhimurium*	LC–MS/MS	Identification of one post-translational modification, lysine acetylation, and of 14 differentially expressed proteins related to antimicrobial resistance	Li et al. 2018
Escherichia coli	2-DE, MALDI-TOF MS, nano HPLC-MS/MS	Identification of differentially expressed proteins under multi drug resistance conditions, highlighting mechanisms involved in resistance (e.g., quorum sensing-mechanisms)	Piras et al. 2012
Escherichia coli	2-DE, MALDI-TOF MS	Upregulation of outer membrane proteins TolC, Tsx and OstA and downregulation of outer membrane proteins MipA, OmpA, FadL and OmpW were found to be downregulated in kanamycin-resistant isolates of the organism	Li et al. 2015
Escherichia coli	2-DIGE, MALDI-TOF/TOF MS, nano LC–MS	Differential expression of proteins linked to oxidative stress response, to DNA protection and to membrane permeability in enrofloxacin-resistant isolates of the organism	Piras et al. 2015
Escherichia coli	2-DE, MALDI-TOF/TOF MS	Overexpression of hydrolase L-asparaginase in a ciprofloxacin-resistant isolate of the organism	Ramos et al. 2016
Mannheimia haemolytica	2-DE, LC/ESI-MS	Administration of chlortetracycline and/or sulfamethazine decreased the expression of leucotoxin	Nanduri et al. 2005
Staphylococcus aureus	2-DE, MALDI-TOF MS	Production of a reproducible and well-resolved reference map of the surface proteins of the organism	Taverna et al. 2007
Staphylococcus aureus	2-DE, MALDI-TOF/TOF MS	Detection of proteins with specific functions (e.g., catalase, superoxide dismutase) found in resistant isolates, indicating that these proteins can protect intraphagocytic bacteria by potentially destroying hydrogen peroxide produced by the phagocyte	Monteiro et al. 2012

Staphylococcus aureus	LC-MS	Detection of 200 differentially expressed proteins in erythromycin and/or oxacillin-resistant isolates of the organim, which can provide new therapeutic approaches for mastitis.	Liu et al. 2016
Staphylococcus aureus	2-DE, LC-MS/MS	Elucidation of mechanisms involved in development of resistance to methicillin by isolates of the organism	Igrejas et al. 2018
Streptococcus spp.	LC-ESI MS/MS	Identification of 134 peptides associated with resistance to antimicrobial agents	Abril et al. 2020

[1] 1-DE SDS-PAGE: One-Dimensional Electrophoresis Sodium Dodecyl Sulphate–PolyAcrylamide Gel Electrophoresis; 2-DE: Two-Dimensional Electrophoresis; 2-DIGE: Two-Dimensional Difference Gel Electrophoresis; HPLC-MS/MS: High Pressure Liquid Chromatography-Mass Spectrometry/Mass Spectrometry; LC-ESI MS/MS: Liquid Chromatography-ElectroSpray Ionization Mass Spectrometry/Mass Spectrometry; LC-MS: Liquid Chromatography-Mass Spectrometry; LC-MS/MS: Liquid Chromatography-Mass Spectrometry/Mass Spectrometry; MALDI TOF: Matrix-Assisted Laser Desorption/Ionization – Time Of Flight; MALDI TOF/TOF: Matrix-Assisted Laser Desorption/Ionization – Time Of Flight/Time Of Flight; MS: Mass Spectrometry.

References

Abril, A.G.; Carrera, M.; Böhme, K.; Barros-Velázquez, J.; Rama, J. R.; Calo-Mata, P.; Sánchez-Pérez, A.; Villa, T.G. Proteomic characterization of antibiotic resistance, and production of antimicrobial and virulence factors in *Streptococcus* species associated with bovine mastitis. Could enzybiotics represent novel therapeutic agents against these pathogens? *Antibiotics (Basel)* **2020**, *9*, 302.

Condell, O.; Sheridan, Á.; Power, K.A.; Bonilla-Santiago, R.; Sergeant, K.; Renaut, J.; Burgess, C.; Fanning, S.; Nally, J.E. Comparative proteomic analysis of Salmonella tolerance to the biocide active agent triclosan. *J. Proteom.* **2012**, *75*, 4505–4519.

Correia, S.; Nunes-Miranda, J.D.; Pinto, L.; Santos, H.M.; de Toro, M.; Sáenz, Y.; Torres, C.; Capelo, J. L.; Poeta, P.; Igrejas, G. Complete proteome of a quinolone-resistant *Salmonella* Typhimurium phage type DT104B clinical strain. *Int. J. Mol. Sci.* **2014**, *15*, 14191–14219.

Igrejas, G.; Correia, S.; Silva, V.; Hébraud, M.; Caniça, M.; Torres, C.; Gomes, C.; Nogueira, F.; Poeta, P. Planning a one health case study to evaluate methicillin resistant *Staphylococcus aureus* and its economic burden in Portugal. *Fr. Microbiol.* **2018**, *9*, 2964.

Li, H.; Wang, Y.; Fu, Q.; Wang, Y.; Li, X.; Wu, C.; Shen, Z.; Zhang, Q.; Qin, P.; Shen, J.; Xia, X. Integrated genomic and proteomic analyses of high-level chloramphenicol resistance in *Campylobacter jejuni*. *Sci. Rep.* **2017**, *7*, 16973.

Li, L.; Wang, W.; Zhang, R.; Xu, J.; Wang, R.; Wang, L.; Zhao, X.; Li, J. First acetyl-proteome profiling of *Salmonella typhimurium* revealed involvement of lysine acetylation in drug resistance. *Vet. Microbiol.* **2018**, *226*, 1–8.

Li, H.; Zhang, D.F.; Lin, X. M.; Peng, X.X. Outer membrane proteomics of kanamycin-resistant *Escherichia coli* identified *MipA* as a novel antibiotic resistance-related protein. *FEMS Microbiol. Lett.* **2015**, *362*, fnv074.

Liu, X.; Pai, P. J.; Zhang, W.; Hu, Y.; Dong, X.; Qian, P. Y.; Chen, D.; Lam, H. Proteomic response of methicillin-resistant *S. aureus* to a synergistic antibacterial drug combination: a novel erythromycin derivative and oxacillin. *Sci. Rep.* **2016**, *6*, 19841.

Monteiro, R.; Vitorino, R.; Domingues, P.; Radhouani, H.; Carvalho, C.; Poeta, P.; Torres, C.; Igrejas, G. Proteome of a methicillin-resistant *Staphylococcus aureus* clinical strain of sequence type ST398. *J. Proteom.* **2012**, *75*, 2892-2915.

Nanduri, B.; Lawrence, M.L.; Vanguri, S.; Pechan, T.; Burgess, S.C. Proteomic analysis using an unfinished bacterial genome: the effects of subminimum inhibitory concentrations of antibiotics on *Mannheimia haemolytica* virulence factor expression. *Proteomics* **2005**, *5*, 4852–4863.

Piras, C.; Soggiu, A.; Bonizzi, L.; Gaviraghi, A.; Deriu, F.; De Martino, L.; Iovane, G.; Amoresano, A.; Roncada, P. Comparative proteomics to evaluate multi drug resistance in *Escherichia coli*. *Mol. Biosyst.* **2012**, *8*, 1060–1067.

Piras, C; Soggiu, A.; Greco, V.; Martino, P.A.; Del Chierico, F.; Putignani, L.; Urbani, A.; Nally, J.E.; Bonizzi, L.; Roncada, P. Mechanisms of antibiotic resistance to enrofloxacin in uropathogenic *Escherichia coli* in dog. *J. Proteom.* **2015**, *127*, 365-376.

Radford, D.; Strange, P.; Lepp, D.; Hernandez, M.; Rehman, M.A.; Diarra, M.S.; Balamurugan, S. Genomic and proteomic analyses of *Salmonella enterica* serovar Enteritidis identifying mechanisms of induced *de novo* tolerance to ceftiofur. *Fr. Microbiol.* **2018**, *9*, 2123.

Ramos, S.; Chafsey, I.; Hebraud, M., Sousa, M.; Poeta, P.; Igrejas, G. Ciprofloxacin sress proteome of the extended-spectrum beta-lactamase producing *Escherichia coli* from slaughtered pigs. *Curr. Proteom.* **2016**, *13*, 285.

Taverna, F.; Negri, A.; Piccinini, R.; Zecconi, A.; Nonnis, S.; Ronchi, S.; Tedeschi, G. Characterization of cell wall associated proteins of a *Staphylococcus aureus* isolated from bovine mastitis case by a proteomic approach. *Vet. Microbiol.* **2007**, *119*, 240–247.

Table S4. Proteomics studies, which were performed in samples of food of animal origin, reviewed in the text of the paper.

Type of sample	Proteomics methodologies applied [1]	Summary of main findings	Reference
Meat samples from cattle	MALDI-TOF MS	Quick species-level identification of bacteria was achieved within 18 to 30 h after collection of the samples, by means of identifying specific biomarkers	Jadhav et al. 2018
Muscle and adipose tissue samples from cattle	LC MS	Identification of changes in 245 proteins, associated with growth of muscle and related to increase in number and volume of muscular and adipose cells, as potential biomarkers for meat quality	Bonnet et al. 2013
Processed meat samples from cattle	MALDI-TOF MS	Exclusive increase of *Enterococcus* genus bacteria, allowing to distinguish the organism from other species of the genus	Sabença et al. 2020
Muscle samples from pigs	LC-MS	Increased rate of the pentose phosphate pathway in pigs	Marrocco et al. 2013
Muscle samples from pigs	2-DIGE, MS	Detection of 16 proteins associated with differences related to two animal phenotypes divergent for water holding capacity	Di Luca et al. 2013
Central nervous system samples from pigs	2-DE	Identification of protein changes according to pre-slaughter handling stress of animals	Arroyo et al. 2013
Muscle samples from sheep	2-DE, MALDI-TOF MS	Identification of chaperone proteins more highly expressed, due to increased use of energetic pathways	Hamelin et al. 2006
Carcass samples from hares	LC-MS/MS	Development of a technique for detection of *Francicella tularensis* in food supplies	Witt et al. 2020
Chicken meat products	LC-MS/MS	Development of a technique for detection and quantification of staphylococcal enterotoxin B	Bao et al. 2012
Edible aquatic products [2]	2-DE, LC-MS/MS	Proteomic profile of *Vibrio parahaemolyticus* from consumable aquatic products	Zhu et al. 2020
Fish [3]	LC-MS/MS	Development of a technique for detection of β-parvalbumin	Carrera et al. 2012
Black tiger prawns	SDS-PAGE, MALDI-Q ToF MS, ESI Q ToF MS	Proteomics profiling of the allergen tropomyosin	Abdel Rahman et al. 2010
Blue mussels	LC-MS	Identification of proteins indicative of pre-harvest contamination	Tedesco et al. 2013
Blue mussels	nano-LC-ESI MS/MS, MALDI-TOF/TOF/MS	Development of processes for depuration of shellfish contaminated with azaspiracid toxins	Nzoughet et al. 2008
Parma dry-cured ham	2-DE, LC–MS/MS	Identification of proteins differentially abundant in pressed or unpressed hams	Paredi et al. 2017
Cow milk	nano LC-MS/MS	Detection of redox and non-redox protein modifications according to oxidative damage in milk products	Clerens et al. 2013
Cow milk	LC-MS/MS	Development of a technique for detection and quantification of ricin, staphylococcal enterotoxin B and *Clostridium perfringens* epsilon toxin	Dupré et al. 2015

Cow milk	LC-MS/MS	Development of a technique for detection of staphylococcal enterotoxin A and staphylococcal enterotoxin B	Andjelkovic et al. 2016
Cow milk, Goat milk	nano LC-MS/MS.	Development of a technique for detection of milk adulteration.	Nardiello et al. 2018
'Grana Padano' cheese	LC–MS/MS	Identification of proteins characterising the stages of ripening and the milk treatment	Soggiu et al. 2013
Feta cheese	LC–MS/MS	Identification of 489 distinct single-gene products characterizing the product	Anagnostopoulos and Tsangaris 2018
Eggs	2-DE, Q-TOF MS	Identification of proteins (ovalbumin, clusterin, ovoinhibitor, ovotransferrin, prostaglandin D2 synthase) that showed significant changes in abundance during eggs storage	Omana et al. 2011
Royal jelly	2-DE, MALDI-TOF MS	Description of the proteome of royal jelly	Scarselli et al. 2005
Seafood products [4]	LC-MS/MS	Development of a technique for detection of β-parvalbumin	Carrera et al. 2012
Drinking water	LC-MS/MS	Development of a technique for detection and quantification of ricin, staphylococcal enterotoxin B and *Clostridium perfringens* epsilon toxin	Dupré et al. 2015

[1] 2-DE: Two-Dimensional Electrophoresis; 2-DIGE: Two-Dimensional Difference Gel Electrophoresis; ESI Q ToF MS: ElectroSpray Quadrupole-Time Of Flight Mass Spectrometry; LC-MS: Liquid Chromatography-Mass Spectrometry; LC-MS/MS: Liquid Chromatography-Mass Spectrometry/Mass Spectrometry; MS: Mass Spectrometry.

[2] *Aristichthys nobilis*, *Ctenopharyngodon idellus*, *Litopenaeus vannamei*, *Mactra veneriformis*, *Oratosquilla oratoria*, *Ostrea gigas* Thunberg, *Paphia undulate*, *Perna viridis*, *Placopecten magellanicus*, *Ruditapes philippinarum*, *Sinonovacula constricta*, *Solen strictus*,.

[3] Angler, Austral hake, Club mackerel, Common seabream, Common sole, Deep-cape hake, Cod, Four-spot megrim, Gilthead seabream, Horse mackerel, Pink cusk-eel, Ray's bream, Salmon, Swordfish, White seabream, Yellowfin tuna.

[4] Cod fillets, frozen surimi, seafood with angler, seafood with hake, seafood with sole.

References

Abdel Rahman, A.M.; Kamath, S.; Lopata, A.L.; Helleur, R.J. Analysis of the allergenic proteins in black tiger prawn (Penaeus monodon) and characterization of the major allergen tropomyosin using mass spectrometry. *Rapid Commun. Mass Spectrom.* **2010**, *24*, 2462–2470.

Anagnostopoulos, A.K.; Tsangaris, G.T. Feta cheese proteins: manifesting the identity of Greece's national treasure. *Data Brief* **2018**, *19*, 2037–2040.

Andjelkovic, M.; Tsilia, V.; Rajkovic, A.; De Cremer, K.; Van Loco, J. Application of LC-MS/MS MRM to determine staphylococcal enterotoxins (SEB and SEA) in milk. *Toxins* **2016**, *8*, 118.

Arroyo, L.; Marco-Ramell, A.; Soler, M.; Pena, R.; Velarde, A.; Sabria, J.; Unzeta, M.; Bassols, A. Neurotransmitter levels and proteomic approach in pig brain: pre-slaughter handling stress and cognitive biases. In *Farm animal proteomics 2013*, Proceedings of the 4th Management Committee Meeting and 3rd Meeting of Working Groups 1, 2 & 3 of COST Action FA1002, Kosice, Slovakia, 25-26 April 2013; de Almeida A, Eckersall D, Bencurova E, Dolinska S, Mlynarcik P, Vincova M, Bhide MR, Eds.; Wageningen Academic Publishers: Wageningen, The Netherlands, 2013; pp. 192-195.

Bao, K.D.; Letellier, A.; Beaudry, F. Analysis of *Staphylococcus* enterotoxin B using differential isotopic tags and liquid chromatography quadrupole ion trap mass spectrometry. *Biomed. Chromatogr.* **2012**, *26*, 1049–1057.

Bonnet, M.; Kaspric N.; Picard. B. Quest for biomarkers of the lean-to-fat ratio by proteomics in beef production. In *Farm animal proteomics 2013*, Proceedings of the 4th Management Committee Meeting and 3rd Meeting of Working Groups 1, 2 & 3 of COST Action FA1002, Kosice, Slovakia, 25-26 April 2013; de Almeida A, Eckersall D, Bencurova E, Dolinska S, Mlynarcik P, Vincova M, Bhide MR, Eds.; Wageningen Academic Publishers: Wageningen, The Netherlands, 2013; pp. 43-44.

Carrera, M.; Cañas, B.; Gallardo, J.M. Advanced proteomics and systems biology applied to study food allergy. *Curr. Opin. Food Sci.* **2018**, *22*, 9–16.

Clerens, S.; Plowman, J.E.; Haines, S.; Dyer, J.M. A mass spectrometric scoring system for oxidative damage in dairy foods. In *Farm animal proteomics 2013*, Proceedings of the 4th Management Committee Meeting and 3rd Meeting of Working Groups 1, 2 & 3 of COST Action FA1002, Kosice, Slovakia, 25-26 April 2013; de Almeida A, Eckersall D, Bencurova E, Dolinska S, Mlynarcik P, Vincova M, Bhide MR, Eds.; Wageningen Academic Publishers: Wageningen, The Netherlands, 2013; pp. 255-257.

Di Luca, A.; Elia, G.; Hamill, R.; Mullen, A.M. 2D DIGE proteomic analysis of early post mortem muscle exudate highlights the importance of the stress response for improved water-holding capacity of fresh pork meat. *Proteomics* **2013**, *13*, 1528–1544.

Dupré, M.; Gilquin, B.; Fenaille, F.; Feraudet-Tarisse, C.; Dano, J.; Ferro, M.; Simon, S.; Junot, C.; Brun, V.; Becher, F. Multiplex quantification of protein toxins in human biofluids and food matrices using immunoextraction and high-resolution targeted mass spectrometry. *Anal. Chem.* **2015**, *87*, 8473–8480.

Hamelin, M.; Sayd, T.; Chambon, C.; Bouix, J.; Bibé, B.; Milenkovic, D.; Leveziel, H.; Georges, M.; Clop, A.; Marinova, P.; Laville, E. Proteomic analysis of ovine muscle hypertrophy. *J. Anim. Sci.* **2006**, *84*, 3266–3276.

Jadhav, S.R.; Shah, R.M.; Karpe, A.V.; Morrison, P.D.; Kouremenos, K.; Beale, D.J.; Palombo, E.A. Detection of foodborne pathogens using proteomics and metabolomics-based approaches. *Fr. Microbiol.* **2018**, *9*, 3132.

Marrocco, C.; D'Alessandro, A.; Rinalducci, S.; Mirasole, C.; Zolla, L. Untargeted metabolomic analyses open new scenarios in post mortem pig muscles: Casertan and Large White. In *Farm animal proteomics 2013*, Proceedings of the 4th Management Committee Meeting and 3rd Meeting of Working Groups 1, 2 & 3 of COST Action FA1002, Kosice, Slovakia, 25-26 April 2013; de Almeida A, Eckersall D, Bencurova E, Dolinska S, Mlynarcik P, Vincova M, Bhide MR, Eds.; Wageningen Academic Publishers: Wageningen, The Netherlands, 2013; pp. 270-273.

Nardiello, D.; Natale, A.; Palermo, C.; Quinto, M.; Centonze, D. Milk authenticity by ion-trap proteomics following multi-enzyme digestion. *Food Chem.* **2018**, *244*, 317–323.

Nzoughet, K.J.; Hamilton, J.T.; Floyd, S.D.; Douglas, A.; Nelson, J.; Devine, L.; Elliott, C.T. Azaspiracid: first evidence of protein binding in shellfish. *Toxicon* **2008**, *51*, 1255–1263.

Omana, D.A.; Liang, Y.; Kav, N.N.; Wu, J. Proteomic analysis of egg white proteins during storage. *Proteomics* **2011**, *11*, 144–153.

Paredi, G.; Benoni, R.; Pighini, G.; Ronda, L.; Dowle, A.; Ashford, D.; Thomas, J.; Saccani, G.; Virgili, R.; Mozzarelli, A. Proteomics of Parma dry-cured ham: analysis of salting exudates. *J. Agric. Food Chem.* **2017**, *65*, 6307–6316.

Sabença, C.; Sousa, T.; Oliveira, S.; Viala, D.; Théron, L.; Chambon, C.; Hébraud, M.; Beyrouthy, R.; Bonnet, R.; Caniça, M.; Poeta, P.; Igrejas, G. Next-generation sequencing and MALDI mass spectrometry in the study of multiresistant processed meat vancomycin-resistant enterococci (VRE). *Biology* **2020**, *9*, 89.

Scarselli, R.; Donadio, E.; Giuffrida, M.G.; Fortunato, D.; Conti, A.; Balestreri, E.; Felicioli, R.; Pinzauti, M.; Sabatini, A.G.; Felicioli, A. Towards royal jelly proteome. *Proteomics* **2005** *5*, 769–776.

Soggiu, A.; Bendixen, E.; Brasca, M.; Morandi, S.; Piras, C.; Bonizzi, L.; Roncada, P. Milk and cheese microbiome for safety and quality of dairy products. In *Farm animal proteomics 2013*, Proceedings of the 4th Management Committee Meeting and 3rd Meeting of Working Groups 1, 2 & 3 of COST Action FA1002, Kosice, Slovakia, 25-26 April 2013; de Almeida A, Eckersall D, Bencurova E, Dolinska S, Mlynarcik P, Vincova M, Bhide MR, Eds.; Wageningen Academic Publishers: Wageningen, The Netherlands, 2013; pp. 262-265.

Tedesco, S.; Mullen, W.; Mischak, H.; Bradshaw, C.; Cristobal, S. Shotgun proteomics in blue mussels exposed to benthic trawler-induced sediment resuspension from a polluted fjord. In *Farm animal proteomics 2013*, Proceedings of the 4th Management Committee Meeting and 3rd Meeting of Working Groups 1, 2 & 3 of COST Action FA1002, Kosice, Slovakia, 25-26 April 2013; de Almeida A, Eckersall D, Bencurova E, Dolinska S, Mlynarcik P, Vincova M, Bhide MR, Eds.; Wageningen Academic Publishers: Wageningen, The Netherlands, 2013; pp. 258-261.

Witt, N.; Andreotti, S.; Busch, A.; Neubert, K.; Reinert, K.; Tomaso, H.; Meierhofer, D. Rapid and culture free identification of *Francisella* in hare carcasses by high-resolution tandem mass spectrometry proteotyping. *Fr. Microbiol.* **2020**, *11*, 636.

Zhu, Z.; Yang, L.; Yu, P.; Wang, Y.; Peng, X.; Chen, L. Comparative proteomics and secretomics revealed virulence and antibiotic resistance-associated factors in *Vibrio parahaemolyticus* recovered from commonly consumed aquatic products. *Fr. Microbiol.* **2020**, *11*, 1453.

© 2021 by the authors. Licensee MDPI, Basel, Switzerland. This article is an open access article distributed under the terms and conditions of the Creative Commons Attribution (CC BY) license (http://creativecommons.org/licenses/by/4.0/).
